# Formation of low‐pressure reaction textures during near‐isothermal exhumation of hot orogenic crust (Bohemian Massif, Austria)

**DOI:** 10.1111/jmg.12744

**Published:** 2023-09-14

**Authors:** Dominik Sorger, Christoph A. Hauzenberger, Fritz Finger, Manfred Linner, Etienne Skrzypek, Simon Schorn

**Affiliations:** ^1^ Institute of Earth Sciences, NAWI Graz Geocenter University of Graz Graz Austria; ^2^ Geoscience Center University of Goettingen Goettingen Germany; ^3^ Department of Environment & Biodiversity University of Salzburg Salzburg Austria; ^4^ Competence Unit Hard Rock Geology GeoSphere Austria Vienna Austria

**Keywords:** Bohemian Massif, Crd‐Spl symplectite, granulite exhumation, isothermal decompression, reaction textures

## Abstract

Two types of aluminous paragneiss from the Loosdorf complex (Bohemian Massif, NE Austria) contain coarse‐grained granulite assemblages and retrograde reaction textures that are investigated to constrain the post‐peak history of the Gföhl unit in the southern Bohemian Massif. Both types have a peak assemblage garnet–biotite–sillimanite–plagioclase–K‐feldspar–quartz–granitic melt ± kyanite ± ilmenite ± rutile, recording peak metamorphic conditions of ∼0.9–1.1 GPa and ∼780–820°C estimated by isochemical phase equilibrium modelling. The first sample type (Ysper paragneiss) developed (i) cordierite coronae around garnet and (ii) cordierite–spinel and cordierite–quartz reaction textures at former garnet–sillimanite interfaces. Calculated chemical potential relationships indicate that the textures formed in the course of a post‐peak near‐isothermal decompression path reaching ∼0.4 GPa. Texture formation follows a two‐step process. Initially, cordierite coronae grow between garnet and sillimanite. As these coronae thicken, they facilitate the development of local compositional domains, leading to the formation of cordierite–spinel and cordierite–quartz symplectites. The second sample type (Pielach paragneiss) exhibits only discontinuous cordierite coronae around garnet porphyroblasts but lacks symplectites. The formation of cordierite there also indicates near‐isothermal decompression to 0.4–0.5 GPa and 750–800°C. This relatively hot decompression path is explained by the contemporaneous exhumation of a large HP–UHT granulite body now underlying the Loosdorf complex. The timing of regional metamorphism in the granulites and the southern Bohemian Massif in general is well constrained and has its peak at ∼340 Ma. Monazite from Loosdorf paragneiss samples yield a slightly younger age of ∼335 Ma. Although the ages overlap within error, they are interpreted to reflect near‐isothermal decompression and exhumation resulting in the formation of the observed reaction textures.

## 1 INTRODUCTION

Granulite facies rocks provide important information about the thermal and chemical evolution of the Earth's lower continental crust. They preserve a wide range of pressure–temperature conditions and chemical compositions and can be found in a variety of orogenic belts that have formed at different times in Earth's history (e.g., Austrheim, 1990; Bohlen, 1991; Ellis, 1987; Harley, 1989). Constraining their pressure–temperature–composition–time (P–T–X–t) evolution gives insights on the evolution and differentiation of the continental crust, as well as the geodynamic and tectonic history of collisional belts (e.g., Bohlen, 1987; England & Thompson, 1986; O'Brien & Rötzler, 2003; Vielzeuf et al., 1990).

The protoliths of granulite facies rocks are initially mostly saturated in H_2_O (e.g., pelites), before undergoing partial melting and melt loss, and may even be rehydrated in the course of their *P–T* history. Retrieving *P–T–t* conditions despite such complex fluid or melt loss/gain events can therefore be challenging. Exhumed granulites usually exhibit a dry bulk rock composition and frequently retain mineral reaction textures, owing to restricted chemical component transport caused by the limited availability of a transport agent like fluid or melt. Typically, reaction texture formation is attributed to post‐metamorphic peak cooling and decompression (e.g., Carson et al., 1997; Clarke & Powell, 1991; Fitzsimons, 1996; Waters, 1991; White & Powell, 2011) but has also been interpreted in terms of prograde reactions related to partial melting (e.g., Greenfield et al., 1998; Pitra & De Waal, 2001; White et al., 2003). Although a change in *P–T* can cause the replacement of one mineral assemblage by another, the spatial arrangement of the texture is rather controlled by relative element (im)mobility (e.g., Gaidies et al., 2017; Korzhinskii, 1959; Svoboda & Fischer, 2013) and the development of small and local effective equilibration volumes (Korzhinskii, 1959; Lanari & Engi, 2017).

While chemical equilibrium is achieved locally, mineral reaction textures represent open chemical systems, which precludes the determination of a bulk domain composition for a given moment in the textures evolution. The presence of minerals in the texture that are not present in the surrounding matrix (e.g., spinel) makes it particularly evident that the reaction texture is not in global equilibrium with the host rock (White & Powell, 2011). As such, the interpretation of reaction textures using equilibrium phase diagrams calculated for bulk compositions—either for the rock or the reaction domain—is limited and unsatisfactory and could lead to erroneous P– T determination. Alternatively, if the texture‐controlling components can be identified, chemical potential diagrams ( μ– μ) can be used to investigate the spatial arrangement of the respective phases within the texture and reconstruct paths in μ– μ space (e.g., Baldwin et al., 2015; Schorn & Diener, 2017; Štípská et al., 2010; White et al., 2008; White & Powell, 2011).

In this study, we use a number of techniques to reconstruct the *P–T–t*‐history of granulite facies metasedimentary rocks from the high‐grade Moldanubian Zone (Gföhl unit) of the Bohemian Massif in Austria. The investigated rocks of the so‐called Loosdorf complex represent a particularly rare example within the Bohemian Massif, where mid‐crustal rocks were found atop a lower‐crustal felsic‐intermediate granulite body (Moldanubian granulite). In other parts of the Bohemian Massif, these Moldanubian granulites frequently occur in association with metapelitic rocks, but these always belong to tectonically lower units, as the Moldanubian granulites usually form the uppermost unit. The metapelitic rocks of the Loosdorf complex exhibit corona and symplectite textures including cordierite coronae around garnet porphyroblasts as well as complementary cordierite–spinel and cordierite–quartz symplectites at former garnet–sillimanite interfaces. Phase equilibrium modelling and thermobarometric calculations are used to derive *P–T* conditions of the metamorphic peak and a subsequent retrograde stage. Chemical potential relationships provide information about the development of reaction textures during a near‐isothermal decompression path. Finally, isotopic and chemical monazite dating provides temporal constraints on the metamorphic evolution. In terms of orogenic evolution, the investigated mineral reaction textures point to a relatively hot exhumation history after peak conditions, which is explained by coeval exhumation with a neighbouring Moldanubian granulite body.

## 2 GEOLOGICAL BACKGROUND

**FIGURE 1:**
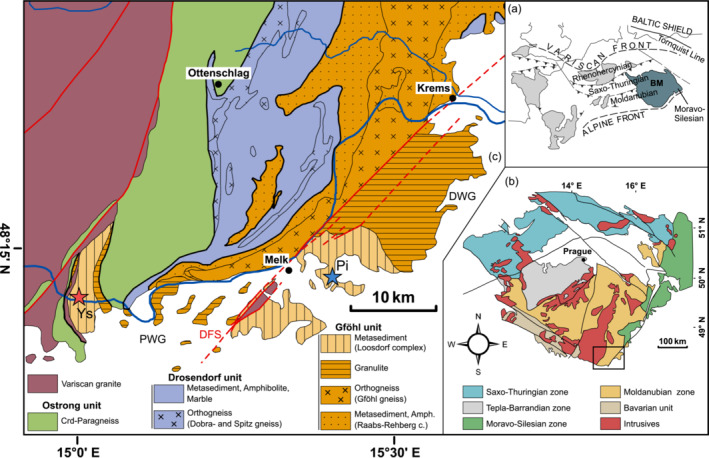
(a) Schematic map of the European Variscan belt, modified after Franke (1989). (b) Simplified map of the Bohemian Massif showing the main tectonic superunits, modified after Franke (2000). (c) Geological map of the Austrian part of the Moldanubian zone, modified after Thiele (1984). The stars indicate the sampled locations in the Ysper valley (Ys) and the Pielach valley (Pi). DFS, Diendorf Fault System; DWG, Dunkelsteinerwald granulite body; PWG, Pöchlarn–Wieselburg granulite body.

The Variscides of central and western Europe have been the focus of numerous studies from which classical structural and tectonic models for the Variscan orogeny and the assembly of Pangea emerged (e.g., Kossmat, 1927; Suess, 1926). The European part of the Variscan orogenic belt extends from the Iberian Peninsula in the west to Poland in the east and consists of several basement blocks. The Bohemian Massif represents the easternmost of these basement blocks (Figure 1a) and covers parts of the Czech Republic, Austria, Germany and Poland. Following Suess ((1903), (1926)) and Kossmat (1927), the Bohemian Massif is traditionally divided into four tectonic superunits: the Saxo‐Thuringian zone, the Moldanubian zone, the Teplá‐Barrandian zone and the Moravo‐Silesian zone (Figure 1b). The whole massif is intruded by numerous granitoid bodies.

The Austrian part of the Bohemian Massif mainly exposes rocks of the high‐grade Moldanubian zone. The major phase of regional metamorphism affected these rocks in the Visean at ∼340 Ma and is thought to correspond to the final collision of the Avalonian (Laurussia)and Armorican (Gondwana) terranes (Finger & Steyrer, 1995; Kroner & Romer, 2013). However, remnants of a ∼370 Ma metamorphic imprint suggest an incipient collision already during the Devonian (Sorger et al., 2020). During the late stage of the Variscan orogeny numerous granitic plutons formed the South Bohemian Batholith, which separates the Austrian Moldanubian zone into a western and eastern part. The western part (the Bavarian unit according to Finger et al. (2007) is characterized by pervasive late Variscan ( ∼320 Ma) LP–HT metamorphism (Kalt et al., 1999; Sorger et al., 2018; Tropper et al., 2006).

East of the batholith, the Moldanubian zone is tectonically divided into the hanging wall Gföhl unit and the underlying Drosendorf and Ostrong units (Fuchs, 1976; Thiele, 1984) (Figure 1c). The Ostrong unit is almost exclusively composed of cordierite bearing, low pressure‐high temperature (LP‐HT) paragneiss (Linner, 1996), with local intercalations of retrogressed eclogite (O'Brien, 1997). The Drosendorf unit comprises paragneiss, marble, quartzite, graphite schist, amphibolite, calc‐silicate and orthogneiss (Fuchs & Matura, 1976). The rocks of the Drosendorf unit experienced medium pressure‐medium temperature (MP‐MT) to HP‐HT Variscan metamorphism (Büttner & Kruhl, 1997; Högelsberger, 1989; Petrakakis, 1986, 1997; Racek et al., 2006; Sorger et al., 2020; Zaydan & Scharbert, 1983).

The Gföhl unit consists of three main lithologies. These are, from bottom to top, the Raabs amphibolite, the migmatic Gföhl orthogneiss and a number of prominent Moldanubian granulite bodies (Thiele, 1984). The latter have a granitic to granodioritic protolith composition and experienced high pressure‐(ultra‐) high temperature (HP–UHT) peak conditions (e.g., Carswell & O'Brien, 1993; Cooke et al., 2000; Cooke & O'Brien, 2001; Schantl et al., 2019). Their equivalents in the Czech Republic are well studied (e.g., Faryad et al., 2010; Kotková et al., 1997; Kotková & Harley, 1999; Tajčmanová et al., 2007; Vrána et al., 2005) and show sporadic evidence of an ultra‐high pressure (UHP) metamorphic stage related to subduction to mantle depths and preceding the high‐temperature stage (Faryad et al., 2013). The amphibolites of the Raabs complex form the structurally lowermost part of the Gföhl unit and have a MORB (mid‐ocean‐ridge‐basalt) geochemical signature (Fritz, 1995) and include some ultrabasite bodies; they are interpreted as part of a former ophiolite (Finger & Steyrer, 1995). Apart from the characteristic mafic rocks, the Raabs complex also exposes metasedimentary rocks. The Raabs rocks record Variscan MP–MT to HP–HT metamorphism, followed by a subsequent stage of isothermal decompression (Petrakakis, 1997).

The so‐called Loosdorf complex (Thiele, 1984), which is the focus of this study, comprises dominantly paragneiss with some intercalations of marble and amphibolite. It is mainly located south of the Danube river, with a smaller continuation north of the Danube along the Ysper valley. The rocks of the Loosdorf complex are only found west and southwest of the Dunkelsteinerwald and Pöchlarn–Wieselburg granulite bodies (DWG and PWG in Figure 1c). Both granulite occurrences originally belonged to a single voluminous Moldanubian granulite body that was displaced by the Diendorf Fault System (DFS in Figure 1c). The western part of the granulite body exhibits a steeply W‐dipping foliation (Scharbert, 1962), suggesting that the Loosdorf complex can be interpreted as the hanging wall part of the granulite and the uppermost part of the Gföhl unit (Thiele, 1984). The granulite bodies further north in Austria and the Czech Republic are also frequently found in the vicinity of metapelitic rocks that have experienced a comparable *P–T* path with MP–MT‐ to HP–HT‐metamorphism and subsequent near‐isothermal decompression. However, these parageneisses typically belong to tectonically lower units and do not lie on top of the granulites, unlike the rocks of the Loosdorf complex.

## 3 PETROGRAPHY AND MINERAL CHEMISTRY

Mineral chemical compositions were quantified with a JEOL JXA‐8530FPlus HyperProbe Electron Probe Microanalyser (EPMA) equipped with an energy‐dispersive and five wavelength‐dispersive spectrometers housed at the Institute of Earth Sciences ‐ NAWI Graz Geocenter, University of Graz. Applied measurement conditions were 15 kV accelerating voltage, 10 nA beam current and a beam diameter of 1–5 µm depending on analysed mineral. For X‐ray element maps of garnet and monazite, the beam current was increased to 150 nA. A set of natural and synthetic standards was used for element calibration. In order to reconstruct the original composition of antiperthitic plagioclase prior to the exsolution, X‐ray element maps of feldspar grains were obtained and quantified using single spot analyses of host and lamellae and the software XMaptools 3.4.1 (Lanari et al., 2014, 2019). To minimize the effects of random cutting through feldspar grains during thin section preparation, this procedure was applied to multiple grains (>5 grains per sample). The petrological elementary tools for Mathematica (PET 7) by Dachs (1998) was used for the calculation of mineral formulae. Small aluminosilicate inclusions (kyanite and sillimanite) in garnet were identified by Raman spectroscopy using a HORIBA Jobin Yvon LabRam‐HR800 Raman micro‐spectrometer. Mineral abbreviations are used after Whitney and Evans (2010), with additional Che for cheralite and Htn for huttonite. Trace element composition of garnet was determined using a laser ablation–inductively coupled plasma–mass spectrometry (LA–ICP–MS) system housed at the NAWI Graz Central Lab for Water, Minerals and Rocks (University of Graz and Graz University of Technology). A spot size of 50 µm, 60 s of ablation and 30 ms dwell time for each mass were used for element analysis with an ESI New Wave 193 Excimer Laser (193 nm wavelength) coupled to a quadrupole Agilent 7500 CX mass spectrometer. Time‐resolved signals were quantified using the data reduction software Glitter (Griffin et al., 2008; Van Achterbergh et al., 2001). Silicon was used as internal calibration element for standardization with NIST SRM 612 (Jochum et al., 2011). The SiO_2_ values used for the respective samples are 38.56 wt% (DR280), 38.39 wt% (DR281), 36.69 wt% (DR265) and 38.01 wt% (DR266).

**FIGURE 2:**
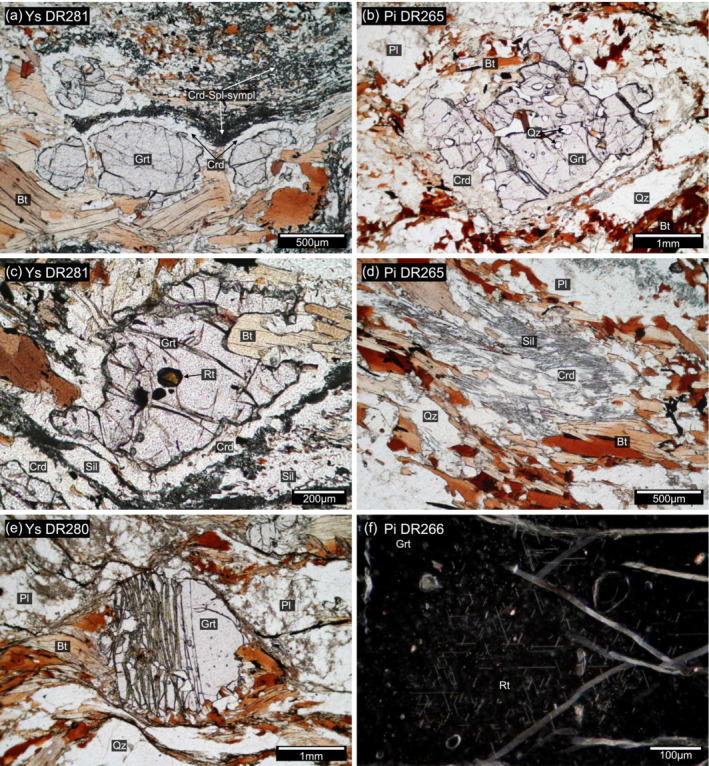
Photomicrographs of investigated samples showing (a, c) monomineralic cordierite corona around garnet separates the grains from cordierite‐spinel symplectites. (b) A highly resorbed garnet porphyroblast with an almost continuous cordierite corona. The cordierite itself is affected by pinitization. (c) Occasionally garnet contains rounded inclusions of brown rutile. (d) Cordierite in the matrix of Pielach samples is often intergrown with sillimanite. (e) Garnet in retrogressed Ysper samples show wide cracks with secondary mineral fillings of biotite and chlorite. (f) Tiny rutile needles with a shape preferred orientation in garnet porphyroblasts of Pielach samples.

Metasedimentary rocks exposed in the Loosdorf complex are typically biotite‐, feldspar‐ and quartz‐rich schist or paragneiss with subordinated garnet, showing a pelitic to psammitic protolith composition. Two types of garnet‐rich varieties from the Ysper valley and the Pielach valley (Figure 1c) were selected for investigation in this study. They are of special interest because of cordierite and cordierite–spinel symplectites replacing garnet (Figure 2a–c), textures that were so far unknown for paragneiss of the Gföhl unit. Samples from both localities display a mineralogy consisting of garnet + biotite + sillimanite + antiperthitic plagioclase ± K‐feldspar + quartz + ilmenite + cordierite ± hercynitic spinel (the latter occurs only in Ysper samples) in variable modal proportions. Additionally, kyanite (only in Pielach samples) and rutile are found exclusively as inclusions in garnet. Accessory minerals are monazite + zircon + apatite + graphite + sulfides. Representative analyses of major mineral phases are shown in Table 1 (garnet, cordierite, spinel and biotite) and Table 2 (feldspars).

**TABLE 1:** Representative garnet, cordierite, spinel and biotite compositions.

Sample	DR281				DR265					280		DR281						DR265	
mineral	Grt							Crd						Spl		Bt			
position	core	ann	rim		core	rim		cor		cor		cor		sym		m		m	gi
SiO_2_	38.58	38.61	38.62		37.80	37.40		48.11		48.46		49.39		0.08		35.45		34.29	34.85
TiO_2_	bld	bld	bld		bld	bld		bld		bld		bld		bld		5.42		5.53	4.78
Al_2_O_3_	21.90	21.96	21.75		21.38	21.28		32.84		32.70		33.06		61.64		16.87		19.14	20.10
Cr_2_O_3_	bld	bld	bld		bld	bld		bld		bld		bld		0.21		0.11		0.11	bld
FeO	28.06	28.21	28.63		32.25	32.63		8.37		8.42		5.69		30.89		15.63		19.04	13.99
MnO	0.63	0.62	0.68		0.78	1.01		0.14		0.33		0.00		0.12		0.00		0.06	0.05
MgO	9.06	8.44	8.81		5.84	5.40		8.05		7.55		10.20		6.07		12.57		8.29	12.08
CaO	1.41	2.07	1.30		1.62	1.65		bld		bld		bld		bld		bld		bld	bld
ZnO	bld	bld	bld		bld	bld		bld		bld		bld		0.75		bld		bld	bld
BaO	nd	nd	nd		nd	nd		nd		nd		nd		nd		0.16		nd9	nd
Na_2_O	bld	bld	bld		bld	bld		0.16		0.22		0.08		bld		0.13		0.15	0.30
K_2_O	bld	bld	bld		bld	bld		bld		bld		bld		bld		9.35		9.26	9.35
F	nd	nd	nd		nd	nd		nd		nd		nd		nd		0.33		0.02	0.11
Cl	nd	nd	nd		nd	nd		nd		nd		nd		nd		bld		0.06	0.06
Total	99.64	99.91	99.79		99.67	99.37		97.67		97.68		98.42		99.76		96.02		95.95	95.67
Oxygen	12	12	12		12	12		18		18		18		4		11		11	11
Si	2.99	2.99	2.99		2.99	2.98		4.99		5.03		5.02		0.00		2.65		2.60	2.58
Ti	0.00	0.00	0.00		0.00	0.00		0.00		0.00		0.00		0.00		0.31		0.32	0.27
Al	2.00	2.00	1.99		1.99	2.00		4.01		4.00		3.96		2.01		1.49		1.71	1.76
Cr	0.00	0.00	0.00		0.00	0.00		0.00		0.00		0.00		0.01		0.01		0.01	0.00
Fe^3+^ ^a^	0.03	0.02	0.03		0.02	0.05		0.00		0.00		0.00		0.00		0.00		0.00	0.00
Fe^2+^	1.78	1.80	1.82		2.11	2.13		0.73		0.73		0.48		0.71		0.98		1.21	0.87
Mn	0.04	0.04	0.05		0.05	0.07		0.01		0.03		0.00		0.00		0.00		0.00	0.00
Mg	1.05	0.97	1.02		0.69	0.64		1.25		1.17		1.54		0.25		1.40		0.94	1.34
Ca	0.12	0.17	0.11		0.14	0.14		0.00		0.00		0.00		0.00		0.00		0.00	0.00
Zn	0.00	0.00	0.00		0.00	0.00		0.00		0.00		0.00		0.02		0.00		0.00	0.00
Ba	0.00	0.00	0.00		0.00	0.00		0.00		0.00		0.00		0.00		0.01		0.00	0.00
Na	0.00	0.00	0.00		0.00	0.00		0.03		0.04		0.02		0.00		0.02		0.02	0.04
K	0.00	0.00	0.00		0.00	0.00		0.00		0.00		0.00		0.00		0.89		0.90	0.88
F	0.00	0.00	0.00		0.00	0.00		0.00		0.00		0.00		0.00		0.08		0.01	0.03
Cl	0.00	0.00	0.00		0.00	0.00		0.00		0.00		0.00		0.00		0.00		0.01	0.01
Total	8.00	8.00	8.00		8.00	8.00		11.02		11.00		11.01		2.99		7.83		7.70	7.77
$X_{\text{alm}}$	0.60	0.6o	0.61		0.71	0.71		‐		‐		‐		‐		‐		‐	‐
$X_{\text{prp}}$	0.35	0.33	0.34		0.23	0.22		‐		‐		‐		‐		‐		‐	‐
$X_{\text{grs}}$	0.04	0.06	0.04		0.05	0.05		‐		‐		‐		‐		‐		‐	‐
$X_{\text{sps}}$	0.01	0.01	0.02		0.02	0.02		‐		‐		‐		‐		‐		‐	‐
$X_{\text{Mg}}$ ^b^	0.37	0.35	0.36		0.25	0.23		0.63		0.62		0.76		0.26		0.59		0.44	0.61

Abbreviations: ann, annulus; bld, below limit of detection; cor, corona texture; gi, garnet inclusion; m, matrix; nd, not determined; sym, symplectite texture.

a Fe^3+^ recalculated after Droop (1987).

b $X_{\text{Mg}}=\frac{\text{Mg}}{\text{Mg}+{\text{Fe}}^{2+}}$.

**TABLE 2:** Representative feldspar compositions.

Sample	DR281					DR265			DR266		DR281		DR265
mineral	Pl										Kfs		
position	mc	mr	cor	sym		m	gi1		gi2		m		m
SiO_2_	60.44	60.40	57.88	44.19		60.66	58.48		61.04		64.20		63.60
Al_2_O_3_	24.39	24.68	26.84	35.30		24.26	25.79		24.63		18.86		18.87
Fe_2_O_3_	bld	bld	bld	0.39		0.14	0.11		0.13		bld		bld
CaO	6.35	7.50	8.24	18.75		5.53	7.30		5.53		0.06		0.07
BaO	bld	bld	bld	bld		bld	bld		bld		0.70		0.58
Na_2_O	7.57	7.26	6.50	0.77		8.44	7.38		8.32		0.97		1.53
K_2_O	0.83	0.35	0.28	bld		0.16	0.30		0.35		15.01		14.63
Total	99.584	100.18	99.73	99.42		99.19	99.36		100.00		99.80		99.28
Oxygen	8	8	8	8		8	8		8		8		8
Si	2.71	2.69	2.59	2.05		2.72	2.63		2.71		2.98		2.96
Al	1.29	1.29	1.42	1.93		1.28	1.37		1.29		1.03		1.04
Fe^3+^	0.00	0.00	0.00	0.01		0.01	0.00		0.00		0.00		0.00
Ca	0.30	0.36	0.40	0.93		0.27	0.35		0.26		0.00		0.00
Ba	0.00	0.00	0.00	0.00		0.00	0.00		0.00		0.01		0.01
Na	0.66	0.63	0.56	0.07		0.73	0.64		0.72		0.09		0.14
K	0.05	0.02	0.02	0.00		0.01	0.02		0.02		0.89		0.87
Total	5.01	4.99	4.99	5.01		5.01	5.02		5.01		5.00		5.02
$X_{\text{ab}}$	0.65	0.62	0.57	0.07		0.73	0.64		0.72		0.09		0.14
$X_{\text{or}}$	0.05	0.02	0.02	0.00		0.01	0.02		0.02		0.90		0.85
$X_{\text{an}}$	0.30	0.36	0.41	0.93		0.26	0.35		0.26		0.00		0.00
$X_{\text{cls}}$	0.00	0.00	0.00	0.00		0.00	0.00		0.00		0.01		0.01

Abbreviations: bld, below limit of detection; cor, corona texture; gi1, garnet inclusion type 1; gi2, garnet inclusion type 2; m, matrix; mc, matrix core; mr, matrix rim; sym, symplectite texture.

### 3.1 Paragneiss from the Ysper valley (samples DR280 and DR281)

**FIGURE 3:**
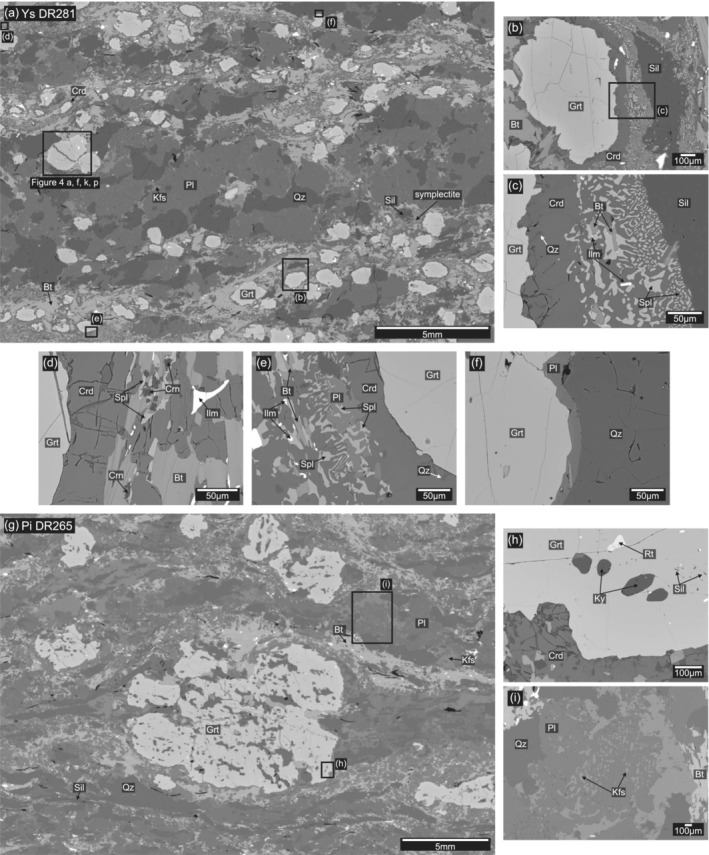
Backscatter electron (BSE) images of (a) the entire thin‐section of sample DR281 showing the general appearance of Ysper samples. (b, c) Monomineralic cordierite coronae and cordierite‐spinel symplectites formed at former garnet‐sillimanite interfaces. (c–e) Besides the main symplectite phases cordierite and spinel, biotite, corundum, anorthite and ilmenite are frequently observed. (f) Monomineralic corona around garnet in quartz‐rich areas. (g) BSE image of the entire thin‐section of sample DR265 showing the different appearance of Pielach samples compared with Ysper. (h) Kyanite, sillimanite and rutile inclusions are observed in the outermost rims of garnet, which are partly replaced by cordierite. (i) Plagioclase in the matrix typically shows antiperthitic exsolution lamellae.

Metapelitic gneiss is exposed in the south of the Yspervalley, close to the mouth of the Ysper creek into the Danube (‘Ys’ in Figure 1c). Eight samples were collected along a ∼20 m wide outcrop, of which two representative ones were selected for further study, one with a pelitic (DR281) and another with a more psammitic (DR280) protolith composition. The gneiss exhibits a migmatitic layering and a distinct foliation defined by biotite, sillimanite and graphite (Figure 3a). Felsic layers mainly consist of coarse grained antiperthitic plagioclase ( ⩽5 mm) and quartz (2–3 mm), with some interstitial K‐feldspar ( ⩽500 µm). Mafic layers consist of biotite + sillimanite + garnet + ilmenite + graphite + sulfides.

The majority of the samples (e.g., DR281) are rich in garnet ( ∼20 vol.%), which forms subhedral, rounded to slightly elongate and millimeter sized (1–3 mm) grains. Garnet grains are typically surrounded by a cordierite corona that separates them from the matrix. Where garnet is in proximity to coarse sillimanite, both are separated by a texture consisting of a cordierite‐quartz symplectite on the garnet side and symplectites of cordierite + hercynitic spinel ± corundum ± anorthite‐rich plagioclase ± biotite ± ilmenite on the sillimanite side (Figures 2a,c and 3a–e). Locally, sillimanite is entirely replaced by symplectites. The textures preferentially occur in biotite–sillimanite rich zones and are limited to quartz‐ and/or feldspar‐absent domains. Garnet embedded in biotite but far from sillimanite, quartz and feldspar commonly shows a monomineralic cordierite corona but lacks spinel‐bearing symplectites. Garnet in direct contact with quartz shows a monomineralic plagioclase corona (Figure 3f) instead of cordierite. Some samples have a more psammitic protolith composition (e.g., DR280, Table 3) and contain significantly less garnet ( ⩽5 vol.%). The few garnet grains are mostly surrounded by an incomplete plagioclase corona, while cordierite occurs only as individual grains scattered near garnet but usually not in direct contact with it.

**TABLE 3:** Representative bulk compositions used for phase equilibrium modelling.

		XRF whole rock compositions (wt.%) of representative samples											
Sample		SiO_2_	TiO_2_	Al_2_O_3_	Fe_2_O_3_	MnO	MgO	CaO	Na_2_O	K_2_O	P_2_O_5_	LOI	Total
DR281		57.12	1.11	18.71	9.17	0.12	4.41	2.28	1.95	2.65	0.12	1.09	98.73
DR280		60.87	1.01	15.91	7.81	0.10	3.80	2.20	2.31	3.32	0.14	1.23	98.89
DR265		59.58	1.01	18.33	8.34	0.09	2.88	1.96	2.48	2.89	0.15	1.29	98.98
DR266		57.78	1.08	19.65	9.58	0.11	3.32	1.40	1.66	2.86	0.13	1.46	99.05

		Normalized modal proportions (mol.%) used for phase equilibrium modelling											
Sample	Figures	SiO_2_	TiO_2_	Al_2_O_3_	FeO	MnO	MgO	CaO	Na_2_O	K_2_O	O	H_2_O	Total
DR281	6a–c	62.81	0.92	12.12	7.59	0.11	7.23	2.50	2.08	1.86	0.28	2.50	100
DR280	6d–f	66.41	0.83	10.23	6.41	0.09	6.18	2.36	2.44	2.31	0.24	2.50	100
DR265	7a–c	65.95	0.84	11.96	6.95	0.09	4.75	2.10	2.66	2.04	0.17	2.50	100
DR266	7d–f	64.49	0.91	12.92	8.05	0.11	5.52	1.47	1.80	2.04	0.20	2.50	100

**FIGURE 4:**
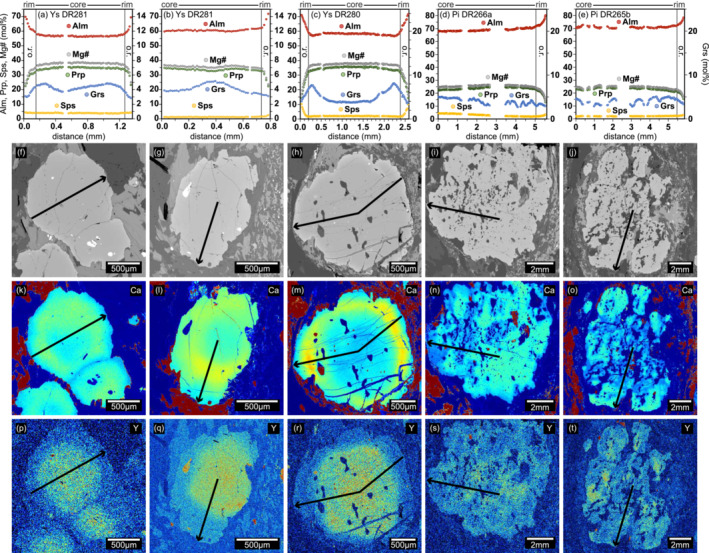
(a–e) Chemical profiles of representative garnet grains along the profile line indicated in panels (f)–(t). X‐ray compositional maps of Ca (k–o) and Y (p–t). o.r., outermost rim.

Garnet is mainly an almandine‐pyrope solid solution with variable grossular and generally low spessartine contents (Alm_57 − 60_Prp_33 − 36_Grs_3 − 8_Sps_2 − 4_, Mg#_35 − 38_) (Figure 4a–c, f–h, k–m and Table 1). The Mg content 
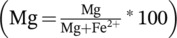
 is relatively constant, except for a distinct decrease at the outermost rim (‘o.r.’ in Figure 4a–c) along with an increase in spessartine (Sps_4 − 10_Mg#_17 − 19_). Garnet usually exhibits a flat grossular profile in the core (Grs_3 − 4_) followed by a grossular‐rich annulus (Grs_5 − 8_) toward the rim and a renewed decrease to values comparable with those in garnet cores. Some garnets show a slight increase in Mg# after the grossular maximum and before Mg# values decrease at the outermost rim (Figure 4b). In samples with a lower abundance of garnet, the grossular‐rich annulus is typically more pronounced (Grs 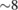; Figure 4c,m) than in garnet‐rich samples (Grs 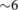; Figure 4a,b,k,l). Garnet hosts rare inclusions of rounded biotite, quartz, rutile, sillimanite, monazite, zircon and sulfides. Most of the inclusions occur in all parts of the grains. Two exceptions are rutile ( ⩽100 µm), which is only observed in grossular‐rich annuli (Figures 2c), and randomly oriented and fine‐grained sillimanite needles that are observed only at garnet rims in sample DR281. Garnet commonly shows irregular fractures without any evidence of secondary mineral fillings. In more retrogressed samples, garnet grains show wider cracks, which are oriented perpendicular to the main foliation and are filled with secondary biotite and chlorite (Figures 2e and 4h).

**FIGURE 5:**
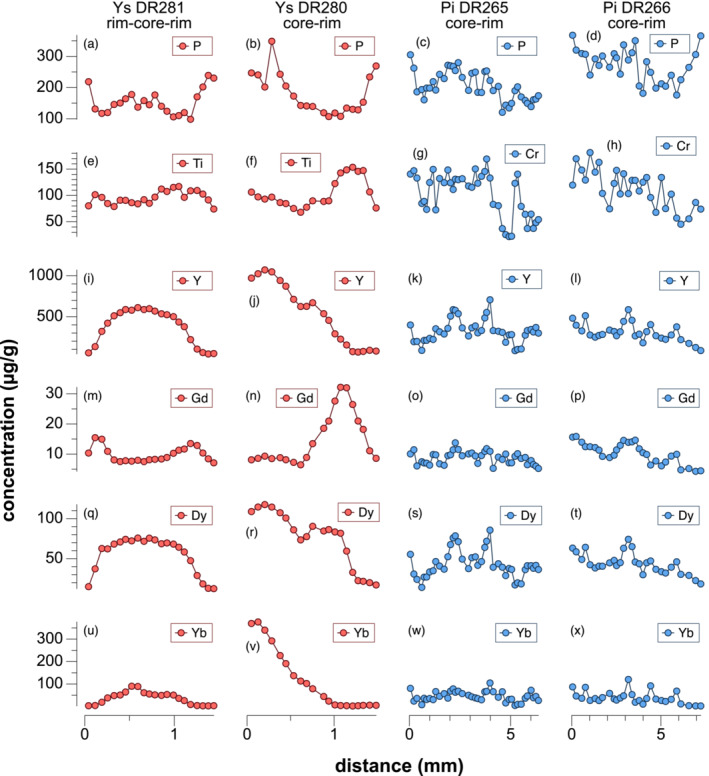
Trace element profiles of garnet porphyroblasts in Figure 4a, c, d, e. LA–ICP–MS profiles were measured along the major element profile lines indicated in Figure 4, where DR281 represents a rim–rim profile and DR280, DR265 and DR266 were measured from core–rim.

Trace‐element zoning patterns show distinct core to rim variations for some elements (Figure 5a–n). Heavy rare‐earth‐elements (HREE) and Y follow a typical Rayleigh fractionation trend with high concentrations in garnet cores, whereas light (La–Sm) and medium (Eu–Dy) rare‐earth‐elements (LREE and MREE) are low in the core. The decrease of HREE toward the rim is locally interrupted by a slight increase (Figure 5j,l). Light to medium REE (Nd–Tb) and Ti notably increase at the Ca‐rich annulus and decrease again toward the rim. Phosphorus shows an opposite trend compared with Ca; it decreases in the Ca‐rich annulus and increases to maximum values at the rim.

Biotite occurs as rare isolated inclusions (10–20 µm) in garnet, as aggregates of single flakes (0.1–1 mm) intergrown with sillimanite in the matrix or as small flakes (10–50 µm) within symplectites. The modal abundance varies from 20 to 30 vol.% between samples. The chemical composition of biotite from different textural positions is relatively uniform within a sample but varies between samples (DR280: Mg#_52 − 54_, DR281: Mg#_59 − 61_). Biotite has significant TiO_2_ contents of ⩽5.42 wt.%, where the highest values were determined from matrix biotite and the lowest from biotite next to garnet and grains from symplectite textures (Table 1). Biotite occasionally shows exsolutions of ilmenite needles along cleavage planes. Small amounts of F (0.19–0.44 wt.%) and BaO ( ⩽ 0.48 wt.%) and no appreciable amounts of Cl were detected.

Cordierite pervades the mafic layers and occurs as thin, (100–200 µm) corona surrounding garnet and as symplectites with hercynitic spinel ± corundum ± anorthite ± biotite (Figures 2a, c and 3a–c). Its composition is homogeneous with Mg#_74 − 77_ in fresh and Mg#_62 − 63_ in retrogressed samples respectively. Pinitization of cordierite occurs only locally in fresh samples, but in more retrogressed samples, cordierite is highly affected by alteration. Cordierite commonly contains traces of MnO ( ⩽0.33 wt.%) and Na_2_O (0.06–0.27 wt.%). Analytical totals between 96.99 and 98.42 wt.% indicate some amounts of volatiles (H_2_O and CO_2_) in cordierite.

Spinel has a dark green colour and occurs as small grains ( ∼1–50 µm) exclusively in symplectites. Its composition is hercynitic (Mg#_23 − 26_) with some ZnO (0.59–1.24 wt.%) and Cr_2_O_3_ (0.16–0.81 wt.%). Some spinel grains have tiny inclusions of corundum and ilmenite, the latter is frequently observed where spinel is in direct contact with biotite.

Plagioclase is, together with quartz, the main constituent of the felsic layers. The large grains (1–5 mm) exhibit relatively coarse ( ⩽100 µm wide) antiperthitic exsolution lamellae and rods. Reintegrated plagioclase has an andesine composition (Table 2) with a minor orthoclase component and shows a slight chemical zoning with an increase in anorthite component at the margins (Ab_62 − 67_An_30 − 37_Or_1 − 5_). Coronitic plagioclase around garnet shows a higher anorthite content (Ab_57 − 60_An_38 − 41_Or_1 − 2_) compared with matrix grains. Small plagioclase grains (10–100 µm) in symplectite textures are almost pure anorthite (Ab_7_An_93_) and locally show tiny spinel inclusions.

K‐feldspar occurs mainly as small interstitial grains ( ⩽500 µm) between plagioclase and quartz or as antiperthitic exsolutions. Matrix grains show a composition with some albite and a minor celsian and anorthite components (Or_89 − 90_Ab_9 − 10_Cn_1 − 2_An_0 − 1_).

### 3.2 Paragneiss from the Pielach valley (samples DR265 and DR266)

Metapelitic gneiss crop out along the Pielach creek (‘Pi’ in Figure 1c). A total of nine samples were collected along a ∼750 m profile, of which two representative samples were selected for detailed investigation. The paragneiss exhibit large garnet porphyroblasts ( ⩽1.2 cm), with a foliation defined by biotite, sillimanite and graphite wrapped around them (Figure 3d). The felsic domains of the matrix mainly consist of antiperthitic plagioclase (1–3 mm), quartz (1–5 mm) and K‐feldspar (0.5–2 mm). Garnet rims are commonly resorbed and replaced by cordierite (Figures 2b and 3e).

Garnet porphyroblasts occur as anhedral to subhedral grains with sometimes highly resorbed rims and irregular cracks filled with secondary biotite and chlorite. Porphyroblasts occur in various grain sizes, and whereas most have a size of ⩽5 mm, a few grains have diameters of up to 1.2 cm. Garnet composition is Fe‐rich with a relatively flat zoning profile (Alm_68 − 72_Prp_21 − 25_Grs_3 − 5_Sps_2 − 4_, Mg#_24 − 27_) (Figure 4d, e, i, j, n, o and Table 1), with the exception of an increase in almandine and spessartine and a decrease in pyrope component and Mg# at the outermost rim (‘o.r.’ in Figure 4d, e). Some grains show a slightly elevated spessartine content in the cores. X‐ray maps (Figure 4n,o) show a patchy Ca distribution observable as an irregular zoning profile of grossular. Garnet grains exhibit irregularly distributed inclusions of mainly quartz and biotite in addition to rutile, ilmenite, plagioclase, kyanite, apatite, monazite and zircon. Additionally, small inclusions of sillimanite are mainly restricted to the rim of garnet porphyroblasts, where they are typically observed together with rutile and kyanite (Figure 3e).Garnet hosts numerous tiny rutile needles (Figure 2f). Therefore, we treat the trace element analyses (mainly Ti, Nb and Ta values) with caution due to the high abundance of inclusions. REE, Y, Cr and P are enriched in the cores of some garnet grains. Yttrium and most of the REEs show a patchy distribution following the zoning pattern of Ca. The zoning profiles have an irregular shape but show a general decreasing trend toward the rim.

Biotite occurs as single flakes (0.1–1 mm) or aggregates in the matrix and constitutes between 15 and 30 vol.% of the samples. It is commonly intergrown with sillimanite and graphite and defines a distinct foliation. In addition, biotite is observed as inclusions ( ⩽500 µm) in garnet poikiloblasts. These are richer in Mg (Mg#_51 − 61_) compared with matrix grains (Mg#_44 − 45_) and have the highest TiO_2_ concentrations of 6.22 wt.%. Uniform F ( ⩽0.20 wt.%) and Cl ( ⩽0.06 wt.%) contents were determined for matrix and inclusion grains.

Cordierite occurs in coronae around garnet porphyroblasts (Figure 2b) and as individual grains intergrown with fine acicular sillimanite in biotite‐rich zones (Figure 2d).Coronae are sometimes discontinuous and only partially replace large garnet grains. Pinitization is common for corona cordierite and less common for matrix grains. The composition is homogeneous with a (Mg#_62 − 64_) and some amounts of MnO (0.9–0.15 wt.%) and Na_2_O (0.11–0.16 wt.%). Analytical totals ranging from 97.03 to 98.59 wt.% indicate some amounts of H_2_O and/or CO_2_ in the cordierite.

Plagioclase is a main constituent of the felsic matrix where it forms coarse grains (1–3 mm) and exhibits wide ( ⩽150 µm) antiperthitic exsolution lamellae and rods (Figure 3f). The reintegrated plagioclase is andesine with a minor orthoclase component (Ab_69 − 75_An_24 − 27_Or_1 − 4_). Two types of plagioclase inclusion can be observed in garnet porphyroblasts. The first is rare inclusions in garnet cores that have a more anorthitic composition than matrix plagioclase (Ab_63 − 66_An_34 − 35_Or_0 − 2_). The second occurs in garnet rims and has a composition comparable to that of matrix grains (Ab 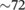
An 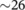
Or 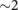).

K‐feldspar occurs as rounded or elongated grains in the matrix (0.5–2 mm) or as exsolutions of antiperthitic plagioclase. The composition is homogeneous with moderate amounts of Na and minor Ba (Ab_14 − 15_Or_84 − 85_Cn_0 − 1_).

## 4 PETROLOGICAL INTERPRETATIONS

### 4.1 Peak metamorphic assemblages

The investigated samples host coarse‐grained assemblages and a metamorphic fabric consisting of melanocratic‐ and leucocratic layers. The former are rich in garnet, sillimanite and biotite, whereas the latter are dominated by quartz and feldspars and are interpreted as leucosomes. Cordierite and spinel—when present—are confined to reaction textures, whereas rutile occurs as inclusions in garnet. As such, we interpret the paragenesis of garnet–biotite–sillimanite–plagioclase–K‐feldspar–quartz–granitic melt ± ilmenite ± rutile as the peak metamorphic assemblage for samples DR280 and DR281 (Ysper paragneiss). Garnet in samples DR265 and DR266 (Pielach paragneiss) additionally contains oriented needles of rutile and inclusions of kyanite and rounded rutile in the close vicinity of sillimanite, all concentrated at the rim of larger grains. These inclusions indicate peak metamorphic conditions, probably close to the kyanite–sillimanite transition.

### 4.2 Garnet zoning

Garnet porphyroblasts in the Ysper paragneiss are characterized by pronounced Ca and trace element zonation (Figure 4 and Figure 5), with a flat Grs profile in the core and a prominent Grs‐rich annulus toward the rim. Pielach paragneiss garnets exhibit an almost homogeneous composition in Fe, Mg and Mn, but an irregular and patchy distribution of Ca, Y and REE (Figure 4 and Figure 5). The preservation of a chemical zoning or at least of the general trend despite high‐grade metamorphism can be explained by the slow diffusion rates of elements like Ca, Y and REE, and/or by a short‐lived metamorphic event. This has been described for other lithologies in that area (e.g., Schantl et al., 2019; Sorger et al., 2020).

A prograde growth zoning was probably eliminated or strongly modified by diffusion under granulite facies peak conditions, such that the observed zonation no longer corresponds to the original zoning profile. The homogeneous profile of Mg# and Mn, observed in all garnets, is likely to be the result of completed diffusive flattening at granulite facies peak conditions. However, in the case of rapid exhumation without significant heating, as assumed for the Loosdorf complex, re‐equilibration would have affected only the peripheral zones of the large garnet grains (Caddick et al., 2010) and likely explains the strong decrease of Mg# at the outermost rim (Figure 4a–e). The garnet composition at peak conditions could then have been preserved or only slightly modified in the more interior parts of porphyroblasts. Therefore, we interpret the Grs‐rich annulus of Ysper garnets and the Grs content at the rims of Pielach garnets (the slightly more interior part in the region of the Mg# plateau) and the homogeneous Mg# plateau in both garnets as the composition close to the peak conditions. The strong decrease of Mg# is then related to retrograde diffusional exchange of Mg and Fe with another ferromagnesian phase like biotite or codierite. Retrograde diffusion, however, probably affects not only the garnet chemistry but also the composition of other phases used for thermobarometry, for example, anorthite content in plagioclase. A modification of the composition of matrix plagioclase would then lead to an underestimation of the obtained pressure conditions.

Taking all this together, we can say that an effect of retrograde diffusion on our *P–T* estimates cannot be ruled out or is even quite likely, which would mean that thermobarometry and thermodynamic modelling provide only minimum estimates. However, as mentioned above, short‐lived retrograde evolution would keep this effect rather small, so our results still represent a close approximation of peak conditions.

### 4.3 Reaction textures

Reaction textures involving coronae and symplectites develop when the exchange of chemical components between reacting minerals is sluggish, causing the formation of local compositional domains (Korzhinskii, 1959; Lanari & Engi, 2017). Due to the Arrhenius‐type relationship between diffusivity and temperature, cooling from the thermal maximum eventually leads to the arrest of textural development, with local equilibrium assemblages and gradients in chemical potential being ‘frozen in’ (e.g., Powell et al., 2019; White et al., 2008). The formation of reaction textures is thus seen as the result of incipient flattening of chemical potentials ‘landscapes’ in an attempt to reach equilibrium at post‐peak conditions, but hampered by limited and variable element mobility (e.g., Powell et al., 2019).

For a given length and time scale of texture formation, components can be grouped in terms of their effective mobility (e.g., White et al., 2008). Highly mobile components such as H_2_O are assumed to be perfectly mobile at the texture scale, implying that their chemical potentials can be assumed as constant and superimposed on the texture. Strongly bonded components, for example, Al_2_O_3_ and SiO_2_, are significantly less mobile—their concentrations and chemical potentials differ but cannot be efficiently equalized across the texture within the time scale of its formation. Conditionally mobile components (e.g., FeO, MgO and CaO) lie between these two end‐members, having variable chemical potentials while being sufficiently mobile to partially equalize across the texture. The latter typically control texture formation and are specified as axes in calculated chemical potential diagrams (e.g., Baldwin et al., 2015; Doukkari et al., 2018; Schorn et al., 2020).

In our samples, reaction textures developed preferentially on garnet and at former garnet–sillimanite interfaces. Assuming components to be respectively effectively (im)mobile for texture characterization, monomineralic coronae form when one component is effectively immobile, while bimineralic symplectites develop in response to two components being effectively immobile—as dictated by the phase rule (Münster, 1970). We therefore interpret the observed textures in terms of a two‐stage process. In this interpretation, the first stage of texture development involved the formation of cordierite coronae on garnet, due to the effective immobility of one component, most likely Al_2_O_3_. As the cordierite corona grew wider, it began to effectively shield garnet from the matrix, thereby hampering further element transport. For isolated garnet grains, texture development stopped at this point.

At former garnet–sillimanite interfaces, gradients in the chemical potentials of FeO, MgO, SiO_2_ and CaO drove transport of these components from garnet toward sillimanite during growth of the cordierite corona. Once this corona grew wide enough there, it caused further component(s) to become effectively immobile at the given time and length scales, leading to the formation of two distinct sub‐domains focused on the garnet and sillimanite sides of the corona. A second stage of texture formation then led to the development of polymineralic symplectites of cordierite–spinel ± corundum ± anorthite ± biotite ± ilmenite on the sillimanite side, and a symplectite of cordierite–quartz on the garnet side of the cordierite corona.

## 5 PHASE EQUILIBRIUM MODELLING

### 5.1 Metamorphic peak and retrograde conditions

In order to reconstruct the *P–T* evolution of paragneiss samples, equilibrium phase diagrams were calculated using thermocalc v3.47 (Powell & Holland, 1988) and an updated version of the internally consistent Holland and Powell (2011) data set (ds63, 2015 update). The activity‐composition (*a–x*) models are those of White et al. (2014a) with updates for Mn bearing phases of White et al. (2014b). The aluminosilicates, quartz, rutile and aqueous fluid (H_2_O) are assumed as pure endmembers. Bulk‐rock chemical compositions (Table 3) were determined by X‐ray fluorescence (XRF) using a Bruker Pioneer S4 XRF housed at the Institute of Earth Sciences ‐ NAWI Graz Geocenter, University of Graz. About 60 international reference samples were used for calibration and reference material GSP‐2 was analysed routinely as a precision monitor. Calculations were performed by transforming the analysed bulk‐rock compositions into the simplified model system MnO–Na_2_O–CaO–K_2_O–FeO–MgO–Al_2_O_3_–SiO_2_–H_2_O–TiO_2_–O_2_ (MnNCKFMASHTO). The presence of graphite and various sulfides and the absence of oxidised phases such as magnetite or hematite in all samples indicate a rather reduced environment during metamorphism. Therefore, the amount of O was set to convert only ∼5 % of total iron to Fe_2_O_3_. The amount of CaO was corrected for the presence of apatite using the analysed amount of P_2_O_5_. The bulk compositions used for phase diagram calculations are provided in Table 3.

The preservation of the granulite facies assemblage and in particular of reaction coronae and symplectites indicates a relatively dry composition. Therefore, we can assume that the analysed bulk‐rock compositions represent those of melt‐depleted residua. The amount of H_2_O used for modelling was set to stabilize the inferred peak mineral assemblage just above the solidus and approximates the modal abundance of hydrous minerals (i.e., biotite) observed in thin section (Diener et al., 2008; Sorger et al., 2020; White et al., 2004). For the four modelled samples (DR281, DR280, DR265 and DR266), an H_2_O content of 2.5 mol.% was required to reproduce the biotite‐content observed in the thin sections.

#### 5.1.1 Ysper paragneiss (sample DR281)

**FIGURE 6:**
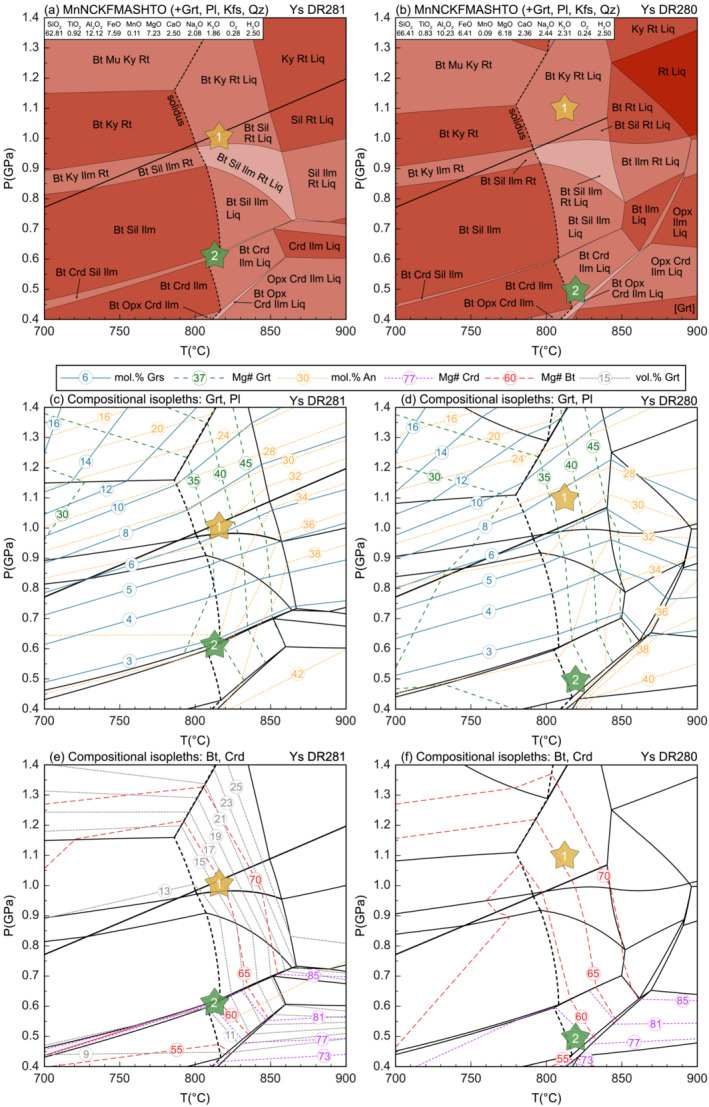
(a, b) Equilibrium phase diagrams of Ysper‐paragneiss samples DR281 and DR280 to model the metamorphic peak (1) and retrograde (2) conditions. The indicated bulk rock compositions refer to mol.%. Calculated isopleths representing (c, d) grossular and Mg# of garnet and anorthite content of plagioclase, and (e, f) Mg# of biotite and cordierite and vol.% of garnet.

The composition of a representative, fresh and garnet‐rich paragneiss sample was used to calculate a phase diagram over 0.4–1.4 GPa and 700–900°C (Figure 6a,c,e). The inferred peak metamorphic assemblage of Grt + Bt + Sil + Kfs + Pl + Rt + Qz + Liq ± Ilm is stable between ∼0.7–1.1 GPa and ∼800–865°C. The occurrence of sillimanite but no kyanite inclusions in garnet rims restricts the maximum pressure below the sillimanite–kyanite transition at ∼1.0–1.1 GPa. The position of the residual solidus indicates temperature conditions of at least 800 to 820°C. To further constrain the *P–T* conditions compositional and/or modal isopleths were calculated for diagnostic minerals. The isopleths representing the composition of the Grs‐rich annulus ( ${\mathrm{Grs}}_{⩽6}$) and the homogeneous Mg# plateau (Mg#_35 − 38_) of garnet as well as the composition of matrix plagioclase cores (An_30 − 32_) intersect at conditions of ∼1.0 GPa and ∼820°C (point ‘1’ in Figure 6a,c,e).

The presence of cordierite coronae and the absence of rutile in the matrix, combined with the composition of biotite and zoned plagioclase, suggest a retrograde stage at different metamorphic conditions. Biotite composition (Mg#_59 − 61_) intersect with the composition of matrix plagioclase rims (An_35 − 37_) and cordierite (Mg#_75 − 77_) at lower pressure conditions of ∼0.5–0.6 GPa (point ‘2’ in Figure 6a,c,e). The temperature conditions for this retrograde stage cannot be unequivocally constrained since the field of the inferred retrograde assemblage (Grt + Bt + Crd + Sil + Kfs + Pl + Ilm + Qz ± Liq) spans a wide temperature range. The elevated Ca concentrations of plagioclase rims (An_35 − 37_) and coronae around garnet (An_38 − 41_), however, still indicate elevated temperatures of ⩾800°C. The spessartine isopleths are relatively uniform (Sps_1 − 2_) over the entire *P–T* range of the diagram and have therefore been omitted. This also applies to the phase diagrams of the other samples described in this section.

#### 5.1.2 Ysper paragneiss (sample DR280)

The second sample of Ysper paragneiss used for phase equilibrium modelling (Figure 6b,d,f) has a slightly different composition (higher Si, Na and K; lower Al, Fe and Mg) and lower modal abundance of garnet. The topology of the diagram is similar to that of sample DR281, except that the occurrence of kyanite/sillimanite is limited to temperatures ⩽850°C. The stability field of the inferred peak assemblage of Grt + Bt + Sil + Kfs + Pl + Rt + Qz + Liq ± Ilm is between ∼0.7–1.1 GPa and ∼800–865°C. The isopleths corresponding to the Grs‐rich annulus (Grs 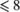), the homogeneous Mg# plateau (Mg#_35 − 38_) of garnet and the core composition of matrix plagioclase (An_29 − 30_) intersect at conditions of ∼1.1 GPa and ∼820°C (point ‘1’ in Figure 6b,d,f). These conditions are just outside the field of the inferred peak assemblage, within the stability field of kyanite. Since neither sillimanite nor kyanite inclusions are found in the garnet rims of sample DR280, the question of whether the peak conditions were on one side or the other of the sillimanite–kyanite transition cannot be definitively determined.

The compositions of biotite (Mg# 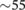) and matrix plagioclase rims/plagioclase coronae around garnet (An_35 − 38_) point to lower pressure conditions of 0.4–0.5 GPa at a temperature of ⩾800°C within the stability field of the inferred retrograde assemblage of Grt + Bt + Crd + Sil + Kfs + Pl + Ilm + Qz ± Liq (point ‘2’ in Figure 6b,d,f).

#### 5.1.3 Pielach paragneiss (sample DR265)

**FIGURE 7:**
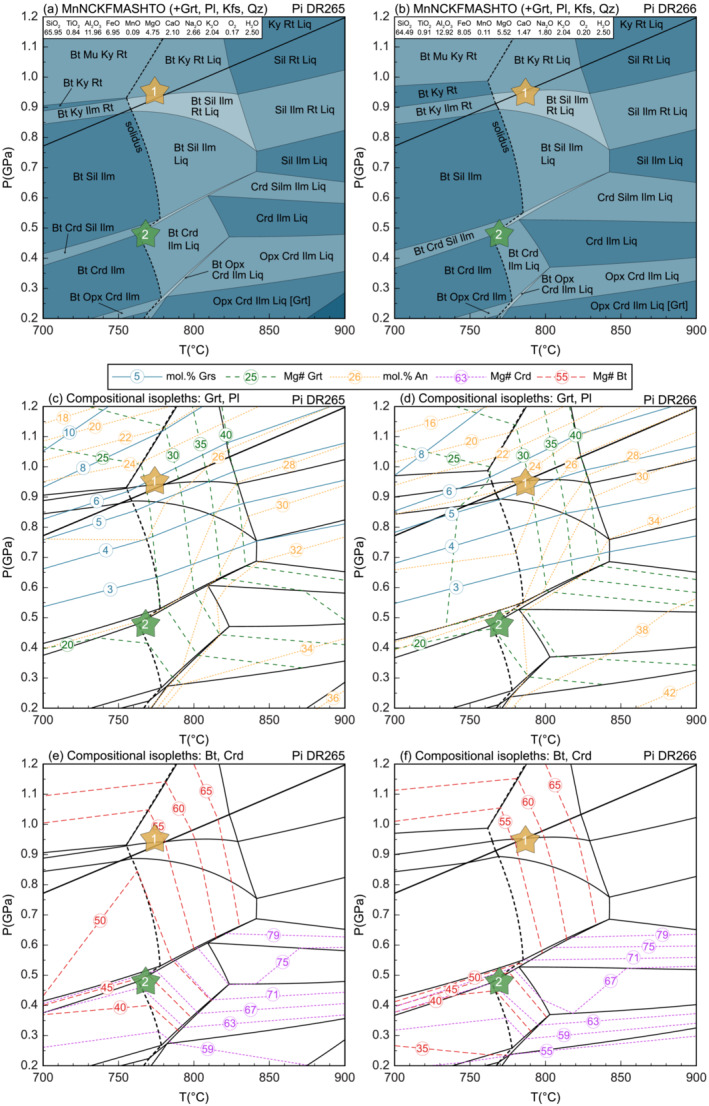
(a, b) Equilibrium phase diagrams of Pielach‐paragneiss samples DR265 and DR266 to model the metamorphic peak (1) and retrograde (2) conditions. The indicated bulk rock compositions refer to mol.%. Calculated isopleths representing (c, d) grossular and Mg# of garnet and anorthite content of plagioclase, and (e, f) Mg# of biotite and cordierite.

A phase diagram was calculated for a representative sample of Pielach paragneiss from 0.2–1.2 GPa and 700–900°C (Figure 7a,c,e). The inferred peak assemblage of Grt + Bt + Ky/Sil + Kfs + Pl + Rt + Qz + Liq ± Ilm is stable between 760 and 840°C. The presence of both kyanite and sillimanite inclusions in garnet rims constrains the peak pressure to be close to the kyanite–sillimanite transition. The position of the residual solidus indicates temperatures of at least 750 to 780°C. Compositional isopleths representing the grossular content of the garnet rim (Grs_5 − 6_) and the homogeneous Mg# plateau (Mg#_24 − 27_) of garnet as well as the composition of biotite inclusions (Mg#_55 − 56_) and plagioclase matrix grains (An_24 − 27_) define *P–T* conditions of ∼0.95 GPa and ∼780°C (point ‘1’ in Figure 7a,c,e).

The compositions of matrix biotite (Mg#_44 − 45_) and cordierite (Mg#_62 − 63_), however, indicate *P–T* conditions of ∼0.4–0.5 GPa at ∼750–800°C within the stability field of the inferred retrograde mineral assemblage of Grt + Bt + Crd + Sil + Kfs + Pl + Ilm + Qz ± Liq (point ‘2’ in Figure 7a,c,e).

#### 5.1.4 Pielach paragneiss (sample DR266)

The phase diagram for a second sample of Pielach paragneiss reveals similar results (Figure 7b,d,f). The inferred metamorphic peak assemblage of Grt + Bt + Ky/Sil + Kfs + Pl + Rt + Qz + Liq ± Ilm is stable at a pressure close to the kyanite–sillimanite transition and temperatureconditions above 760°C  indicated by the position of the residual solidus. The intersection of compositional isopleths corresponding to garnet (Grs_4 − 6_Mg#_24 − 27_), biotite inclusions (Mg#_59 − 61_) and matrix plagioclase (An_24 − 26_) yield ∼0.95 GPa and ∼790°C (point ‘1’ in Figure 7b,d,f).

The presence of cordierite as part of the inferred retrograde assemblage of Grt + Bt + Crd + Sil + Kfs + Pl + Ilm + Qz ± Liq points to lower pressure conditions of ∼0.4–0.5 GPa. This correlates well with the analysed compositions of matrix biotite (Mg#_44 − 45_) and cordierite (Mg#_63 − 64_) (point ‘2’ in Figure 7b,d,f).

### 5.2 Retrograde reaction texture evolution

The retrograde evolution is further constrained using calculated chemical potential relationships. Phase equilibrium modelling indicates decompression from ∼1.0–1.2 GPa to 0.4–0.6 GPa at 800–850°C without significant attendant cooling (Figures 6 and 7). As such, we assume isothermal decompression at the inferred thermal maximum. The development of compositional domains was likely caused by the loss of fluid and/or melt at peak metamorphic conditions; the formation of retrograde textures in granulites is in fact commonly ascribed to cooling/decompression following the last event of melt extraction but still at suprasolidus conditions (White & Powell, 2011). The loss of the majority of melt as transport medium greatly reduces the transport of elements, which favours the development of chemical potential gradients across local compositional domains (Korzhinskii, 1959; Thompson, 1959). The last portions of melt are then either consumed in texture‐forming reactions or finally crystallise when the solidus is crossed (White & Powell, 2011). At this point, intercrystalline exchange slows down significantly and texture formation is halted.

The main texture involves garnet, sillimanite, cordierite, quartz, spinel ± corundum ± anorthite. When ignoring the latter and minor components such as ZnO and Cr_2_O_3_ for simplicity, all phases can be modelled in the five‐component FeO–MgO–Al_2_O_3_–SiO_2_–H_2_O (FMASH) model system. For the modelling, H_2_O is assumed to be perfectly mobile and its chemical potential ( $\mu_{H_{2}O}$) is taken as constant and superimposed over the scale of texture formation. We chose $\mu_{H_{2}O}$ = −400 kJ mol^−1^, the value calculated at peak *P–T*. The texture can be represented in μFeO– μMgO‐space by assuming Al_2_O_3_ as effectively immobile component during formation of the cordierite corona, whereas SiO_2_ is taken to be additionally effectively immobile during development of the cordierite–spinel and cordierite–quartz symplectites. The texture assemblage is univariant in the FMASH chemical system and is represented by the reaction:

(1) $$Grt+Sil+\mu H_{2}O_{\left(melt\right)}⟹Crd+Spl+Qz$$

where μH_2_O represents the H_2_O in the melt phase. Reaction (1) occurs at ∼0.43 GPa and 850°C (White et al., 2014a), which is shifted to slightly higher temperature compared with our estimated peak conditions due to the simplified model system. Whereas the calculated chemical potential relations depend on the chosen P– T– $\mu_{H_{2}O}$ values at which the diagrams are calculated, their absolute values are subordinate compared with the general diagram topology which is controlled by the underlying phase relations (Schorn & Diener, 2017; White et al., 2008).

**FIGURE 8:**
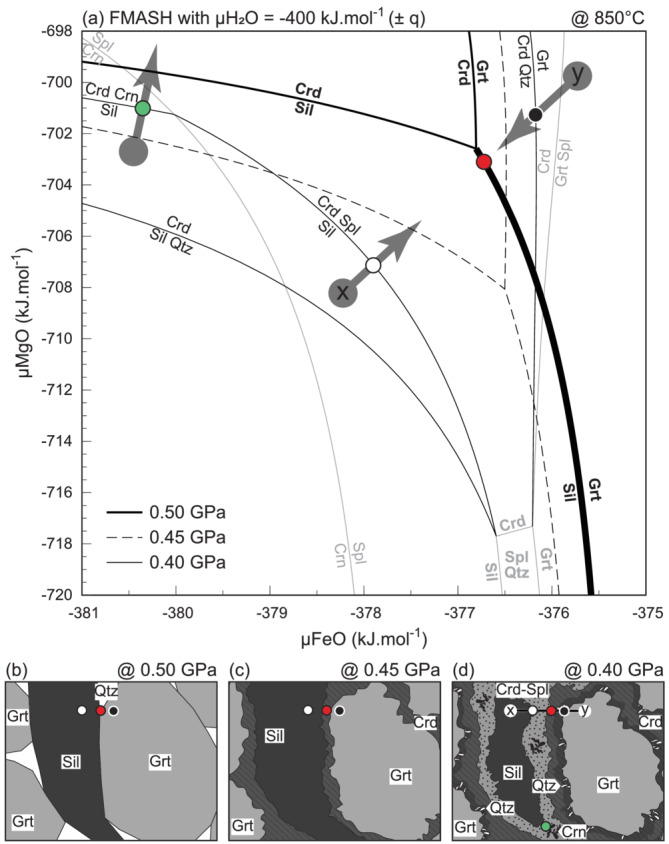
(a) Chemical potential relations calculated for mineral textures observed in Ysper paragneiss sample DR281. (b–d) Sketch showing the two‐step formation of the observed textures. Symbols in the diagrams refer to the respective position in the images.

The calculated chemical potential relationships are presented in Figure 8a. Prior to texture formation (0.50 GPa in Figure 8a), garnet coexists with sillimanite and quartz (red dots and thick line in Figure 8a,b) as part of the inferred peak metamorphic assemblage (Figure 8b). Similarly, the black and white dots in Figure 8a respectively lie within the garnet and sillimanite fields (Figure 8b). Decompression causes all the lines to shift toward lower μFeO– μMgO (dashed lines in Figure 8a), with the calculated phase relations sweeping through the investigated μFeO– μMgO‐space. As long as the pressure lies above the intersection of reaction 1 at the chosen temperature (i.e., ∼0.43 GPa and 850°C), the diagram topology remains unchanged. If transport is not able to maintain equilibrium during this shift, μFeO– μMgO pairs that previously defined the coexistence of garnet–sillimanite–quartz, now partially fall within the stability field of cordierite, and cordierite coronas will form along sillimanite–garnet interfaces (red dots in Figure 8a,c). During initial growth, μSiO_2_ was able to still equalize across the cordierite corona, which is modelled by taking quartz in excess during the first decompression step (Figure 8a).

Further decompression below the pressure where reaction 1 is intersected causes a topological inversion of the calculated chemical potential relationships (thin lines in Figure 8a). Now, sillimanite and garnet no longer coexist (right‐hand side of reaction 1); instead, cordierite and spinel can now stably coexist (Figure 8a). Growth of the cordierite corona continues until it reaches a thickness that is in excess of the transport range of SiO_2_, at which point SiO_2_ becomes effectively immobile in addition to Al_2_O_3_ and bimineralic symplectites begin to grow. On the sillimanite side, a symplectite of cordierite–spinel forms (white dots in Figure 8a,d), whereas a cordierite–quartz symplectite develops on the garnet side (black dots in Figure 8a,d). As these symplectites join the previously formed coronae of cordierite on the appropriate location of former interfaces, the texture is complete and corresponds to that observed in thin section: sillimanite | cordierite–spinel | cordierite | cordierite–quartz | garnet (x–y in Figure 8). Depending on the locally dominant gradients in μFeO– μMgO– μSiO_2_, the symplectite involves spinel or corundum in addition to cordierite (green dots on Figure 8a,d).

## 6 THERMOBAROMETRY

Several thermometers and barometers were used to further constrain *P–T* conditions for Ysper (DR280 and DR281) and Pielach (DR265 and DR266) paragneisses, which are summarized in Table 4. For pressure or temperature dependent methods, estimates from phase equilibrium modelling were used for calculations.

**TABLE 4:** Summarized results from thermobarometric calculations.

ZiR (°C)									GASP (GPa)	
K20	T07	F07	Z04		TiB (°C)		2Fsp (°C)		K09	TWQ
Ysper paragneiss										
Garnet core										
‐	‐	‐	‐		‐		‐		‐	‐
Grs‐rich annulus, Rt inclusions and Pl cores										
662–790	679–792	673–786	765–914		‐		‐		0.90–1.07	0.80–0.90
Matrix phases										
‐	‐	‐	‐		720–781		812 ± 10		‐	‐
Symplectite										
‐	‐	‐	‐		694–762		‐		‐	‐
Pielach paragneiss)										
Garnet and related inclusions										
709–808	721–807	715–801	824–933		767–790		‐		‐	‐
Garnet rim and matrix phases										
‐	‐	‐	‐		727–764		785 ± 11		0.98–1.16	0.98–1.01

*Note*: K20, (Kohn, 2020); T07, (Tomkins et al., 2007); F07, (Ferry & Watson, 2007); Z04, (Zack et al., 2004) K09, (Koziol, 1989); TWQ, Wintwq v2.36 (Berman, 1991).

Zirconium‐in‐rutile (ZiR) thermometry performed with EPMA was applied for rutile inclusions in garnet. Temperature calculations following Kohn (2020), Tomkins et al. (2007) and Ferry and Watson (2007) produced almost identical results, whereas the calibration of Zack et al. (2004) yields temperatures that are 100–130°C higher and therefore not considered for further interpretation. Zirconium contents in rutile from Ysper samples range between 429 and 1384 µg/g resulting in calculated temperatures of 662–792°C. Rutiles from Pielach samples have Zr contents of 681–1599 µg/g equivalent to a temperature range of 709–808°C. The analytical uncertainty of Zr values of 3 to 5 % (relative 2 σ) results in an absolute temperature uncertainty of ± ∼10°C.

Titanium‐in‐biotite (TiB) thermometry applying the calibration of Henry et al. (2005) was used for calculations with biotite from the matrix (Ysper and Pielach), embedded in garnet (only in Pielach) or in symplectites (only in Ysper). Matrix biotite from Ysper samples yields a temperature in the range 720–781°C, with the lowest values coming from biotite surrounding garnet porphyroblasts. Biotite from symplectites corresponds to similar, but slightly lower values of 694–762°C. For biotite inclusions in garnet from Pielach samples a temperature of 767–790°C was obtained, matrix grains gave slightly lower values of 727–764°C. Measured Ti values have an analytical uncertainty of 4 to 6 % (relative 2 σ) resulting in ± 7–9°C.

Two‐feldspar‐thermometry (2Fsp) was applied to K‐feldspar–plagioclase pairs following the method proposed by Kroll et al. (1993) with the thermometer codes of Benisek et al. (2004) and the interaction parameters of Benisek et al. (2010). The reintegrated composition of antiperthitic plagioclase was used together with K‐feldspar grains from the matrix. Errors were calculated according to Benisek et al. (2004). Feldspars from Ysper samples yielded a temperature of 812 ± 10°C; those of Pielach samples a temperature of 785 ± 11°C.

Garnet‐Al_2_SiO_5_‐SiO_2_‐plagioclase (GASP) barometry was used to determine peak pressure conditions using the Grs‐rich annulus of garnet (Ysper samples) or the garnet rim (Pielach samples) together with the composition of matrix plagioclase cores. To minimize any retrograde effects on the calculated pressure, the garnet rim composition in Pielach samples was taken from the edge of the Mg# plateau and not from the outermost rim showing a strong decrease in Mg#. We used two different approaches for calculations: (i) following the method of Koziol (1989) and (ii) the software package Wintwq v2.36 (Berman, 1991) using an updated version of the internally consistent Berman (1988) dataset (December 2006) with the solution models of Fuhrman and Lindsley (1988) for plagioclase and Berman and Aranovich (1996) for garnet. Although both methods produce comparable results, the pressure values obtained from Wintwq are slightly lower compared to that obtained using the calibration of Koziol (1989). Determined pressure conditions for Ysper paragneiss are 1.03 ± 0.05 GPa and 1.09 ± 0.09 GPa and for Pielach samples 1.0 ± 0.05 GPa and 1.07 ± 0.09 GPa.

## 7 MONAZITE GEOCHRONOLOGY

Monazite geochronology on the four samples was conducted with two different methods: (i) isotopic U–Pb dating with LA–MC–ICP–MS (laser ablation–multi collector–inductively coupled plasma–mass spectrometry) and (ii) chemical Th‐U‐total Pb dating using EPMA.

In‐situ U–Pb measurements were done on polished thin sections using a ESI New Wave 193 Excimer Laser (193 nm wavelength) coupled to a Nu Plasma II mass spectrometer at the NAWI Graz Central Lab for Water, Minerals and Rocks (University of Graz and Graz University of Technology). Laser conditions were 4 Hz repetition rate, 15 s ablation time, a fluence of 3.2 J/cm^2^ and a spot diameter of 10 µm. Backscatter electron images and X‐ray compositional maps taken on a JXA‐8530FPlus HyperProbe were used to select analysis points. Twelve analyses of unknown samples were bracketed by three of monazite reference material 44069 (Aleinikoff et al., 2006) and three of NIST SRM 610 glass (Jochum et al., 2011). Among the unknowns, we routinely analysed Moacyr (‘Paraiso’ type; published ^206^Pb/^238^U age: 510 ± 6 Ma; weighted mean ^206^Pb/^238^U age this study: 511 ± 2 Ma; Gonçalves et al., 2016) and Ryoke monazites (EY42M and EY135A; published ^206^Pb/^238^U zircon ages from the same sample: 94 ± 3 Ma and 98 ± 1 Ma; weighted mean of this study: 94 ± 1 and 99 ± 1 Ma; Skrzypek et al., 2016) as secondary reference materials. A detailed description of analytical conditions is provided in Table S1. For data reduction and age calculation, we used Iolite v3.71 (Paton et al., 2010, 2011) with the U_Pb_Geochron4 data reduction scheme, without applying any common lead correction. Weighted mean ages (95% confidence) and concordia diagrams were calculated using IsoplotR (Vermeesch, 2018). The discordance of single dates was calculated using the concordia distance method (Vermeesch, 2021). All analyses with a discordance ⩽1% are referred to as concordant. Only these dates are used to calculate weighted mean ^206^Pb/^238^U ages. Discordant dates are included as light grey bars or ellipses in weighted mean and concordia diagrams.

Th–U–total Pb dates, REE and trace elements were obtained with a high spatial resolution of 3–5 µm. For chemical dating, the EPMA was operated with a high beam current of 150 nA at 15 kV and increased counting times for elements of interest in order to increase precision. The M α lines of Th and U were analysed with a PETL crystal, and that of Pb with a PETH crystal. Typical counting times were 300 s on peak and background for Pb and 150 s for Th and U. Metallic standards were used for calibration of Th and U, natural crocoite (PbCrO_4_) for Pb and synthetic phosphates for Y and REE. Ages were calculated following Montel et al. (1996) and resulted in relative errors of 5–10% (2 σ). Accuracy was monitored by routinely analysing the reference monazites Elk Mountain (published ^207^Pb/^206^Pb age: 1391–1404 Ma; weighted mean this study: 1391 ± 6 Ma; Peterman et al., 2012), RW‐1 (published ^207^Pb/^235^U age: 904 ± 1 Ma; weighted mean Th–U–total Pb age this study: 917 ± 7 Ma; Ling et al., 2017); Gföhl gneiss (published ^206^Pb/^238^U zircon age: ∼340 Ma; weighted mean Th–U–total Pb age this study: 338 ± 2 Ma; Friedl et al., 2004) and an ‘ekanite’ from Sri Lanka (published ^207^Pb/^206^Pb age: 562 ± 1 Ma; weighted mean Th–U–total Pb age this study: 563 ± 4 Ma; Nasdala et al., 2017). The total dataset of analysed monazite is available in Table S2. Weighted mean ages (95 % confidence) were calculated using IsoplotR (Vermeesch, 2018).

### 7.1 Monazite characterization

**FIGURE 9:**
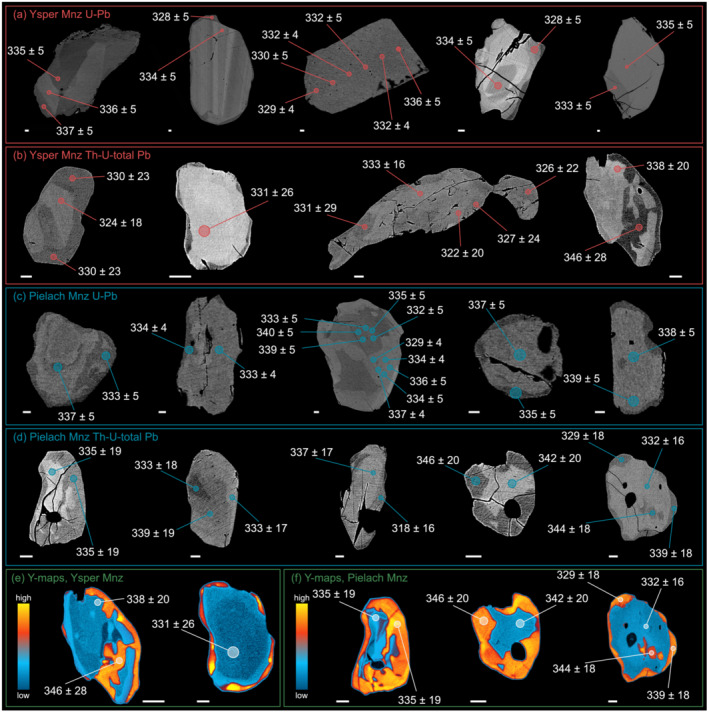
(a–d) BSE images of analysed monazite grains, with locations of ^206^Pb/^238^U or Th–U–total Pb dates (in Ma, with 2 σ error). (e, f) X‐ray compositional maps for Y show typically Y‐poor monazite cores and Y‐rich rims or fringes. The scale bar always represents 10 µm.

The general appearance and composition of monazite are very similar for all analysed samples and are therefore described together here, taking into account all results from the Ysper and Pielach samples. Figure 9 shows the position and respective dates of analysed spots of representative monazite grains.

(Ce‐)Monazite occurs as anhedral grains 20 to 200 µm in size and is generally rich in Ce_2_O_3_ (26.68–31.10 wt.%), La_2_O_3_ (12.06–15.99 wt.%) and Nd_2_O_3_ (11.70–14.67 wt.%), with minor amounts of Pr_2_O_3_ (3.00–3.57 wt.%), Sm_2_O_3_ (1.48–2.57 wt.%), Gd_2_O_3_ ( ⩽1.03 wt.%) and Dy_2_O_3_ (0.87–2.15 wt.%). Monazite appears in three different textural positions: (i) inclusions in garnet, (ii) in the matrix and (iii) in mineral reaction textures. Often the grains show distinct chemical zoning, which is visible on BSE images as bright cores and dark rims or fringes (Figure 9). Bright zones show the highest ThO_2_ ( ⩽6.15 wt.%) and UO_2_ ( ⩽2.23 wt.%) contents, but comparably low Y_2_O_3_ values. Dark fringes, in contrast, are characterized by high Y_2_O_3_ ( ⩽3.63 wt.%) contents (Figure 9e,f) and tend to have lower ThO_2_ and UO_2_. Rare monazite inclusions in garnet porphyroblasts usually lack these Y‐rich fringes. No differences were found in the chemical composition of matrix grains and monazite in reaction textures.

Relatively higher CaO (0.66–1.35 wt.%) and lower SiO_2_ (0.06–0.41) contents indicate that Th and U were predominantly incorporated into the monazite structure by the cheralite substitution $\left[{\left(\text{Th, U}\right)}^{4+}+{\text{Ca}}^{2+}\;\Leftrightarrow \;2{\text{REE}}^{3+}\right]$ and only weakly via the huttonite exchange $\left[{\left(\text{Th, U}\right)}^{4+}+{\text{Si}}^{4+}\;\Leftrightarrow \;{\text{REE}}^{3+}+P^{5+}\right]$ (Cuney & Friedrich, 1987; Foerster, 1998): Mnz_89 − 94_Che_5 − 10_Htn_0 − 2_.

### 7.2 Results of U–Pb geochronology

**FIGURE 10:**
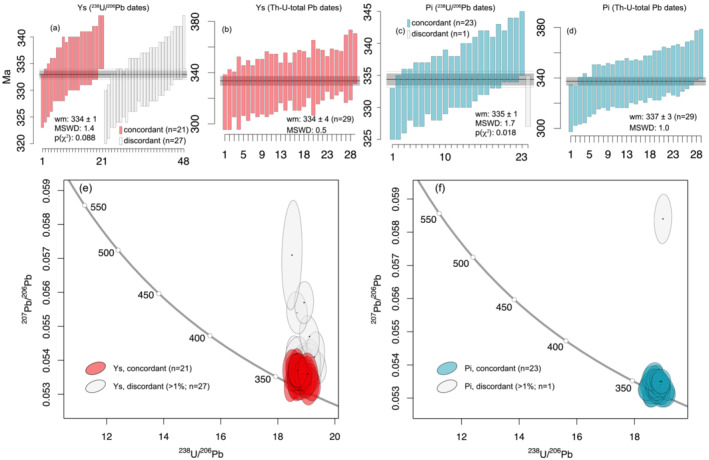
Results from (a, c) isotopic U–Pb and (b, d) chemical Th–U–total Pb monazite geochronology of (a, b) Ysper (Ys) and (c, d) Pielach (Pi) paragneiss samples. The black line indicates the calculated weighted mean age, the grey field the 95% confidence interval. (e, f) Tera‐Wasserburg concordia diagrams for monazite U–Pb data shown in panels (a) and (c), respectively.

U–Pb isotopic measurements yielded ^206^Pb/^238^U dates ranging between 328 ± 5 Ma and 339 ± 5 Ma for Ysper samples ( n = 48) and between 329 ± 4 and 340 ± 5 Ma for Pielach samples ( n = 24) with a weighted mean age of 334 ± 1 Ma and 335 ± 1 Ma, respectively (Figure 10a,c). A correlation of the obtained dates with the textural position of monazite could not be asserted. A total of 27 dates from Ysper samples have a discordance ⩾1% and point to the presence of common Pb (Figure 10e). For Pielach monazites, there is only a single date that shows a discordance ⩾1%. Neither profiles measured across larger monazite grains (Figure 9a, grain 3) nor repeated measurements within different compositional domains (Figure 9c, grain 3) could reveal a systematic trend or relationship between the ^206^Pb/^238^U dates and the chemical composition within the analytical uncertainty of the employed methods.

### 7.3 Results of Th–U–total Pb geochronology

Results of Th–U–total Pb measurements define a single population with weighted mean ages of 334 ± 4 Ma (317 ± 22 to 351 ± 20 Ma, n = 29) for Ysper samples and 337 ± 3 Ma (316 ± 19 to 359 ± 20 Ma, n = 29) for Pielach samples (Figure 10b,d). The typical uncertainty on a single analysis is ∼15–20 Ma (2 σ). In both Ysper and Pielach samples, monazite inclusions are present in garnet but cannot be distinguished from matrix grains in terms of composition or date. Even with the higher spatial resolution of the EPMA measurements, we could not detect a systematic correlation between the composition and the Th–U–total Pb data of the different monazite zones.

## 8 DISCUSSION

**FIGURE 11:**
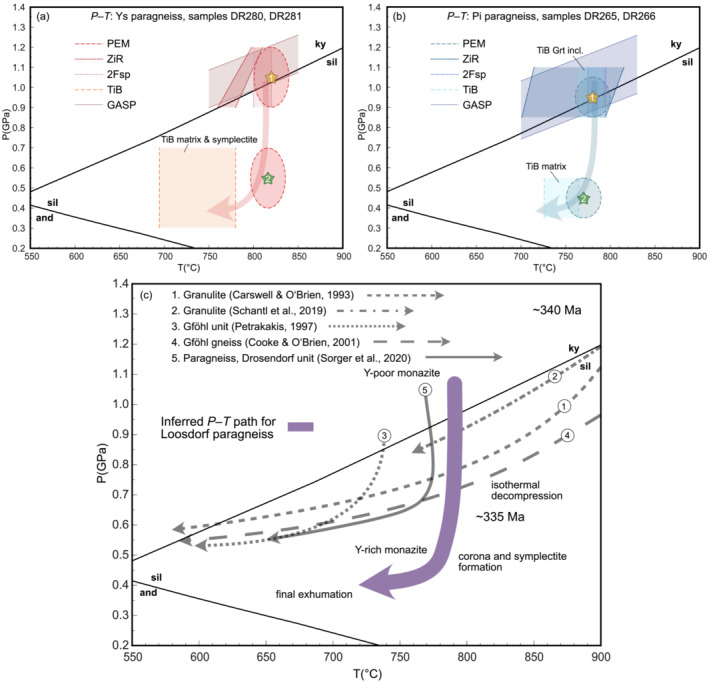
Compiled results of thermodynamic modelling and thermobarometric calculations, and the reconstructed *P–T* path of (a) Ysper and (b) Pielach paragneisses. (c) Proposed *P–T* paths of Loosdorf paragneiss in comparison to the retrograde parts of proposed *P–T* paths from other lithologies of the (1–4) Gföhl unit and a (5) paragneiss from the Drosendorf unit. The ∼340 Ma age indicates the proposed age of the HP granulite facies metamorphism in the Moldanubian zone, and the ∼335 Ma age is dominant age obtained from monazite in the Loosdorf complex.

Granulite facies paragneisses from the Loosdorf complex exhibit coronae and symplectite reaction textures, predominantly where chemically zoned garnet was in contact with sillimanite. Isochemical phase diagrams were used in combination with thermobarometric calculations to constrain the peak metamorphic conditions and the subsequent retrograde evolution. Chemical potential relationships were additionally interrogated to explain the formation of mineral reaction textures. Two different approaches of monazite geochronology, namely, isotopic U–Pb and chemical Th–U–total Pb dating, yielded identical ages of ∼335 Ma. The compilation of *P–T* estimates and the reconstructed *P–T–t* path are summarized in Figure 11.

### 8.1 P–T evolution and garnet zoning

#### 8.1.1 Ysper paragneiss

Due to high temperature modifications of the compositional profile of garnet, as well as the absence of mineral inclusions representative for an early stage of metamorphism, it is not possible to make quantitative statements about the prograde part of the *P–T* path. However, by combining thermodynamic modelling with various exchange thermobarometers and petrographic observations, we are able to reconstruct the metamorphic peak conditions and the retrograde *P–T* path (Figure 11).

The typical Ca‐zoning of Ysper garnets exhibits a flat profile in the core and a grossular‐rich annulus toward the rim followed by a renewed decrease. The increase in grossular coincides with the first appearance of rutile inclusions, both indicative of a potential pressure increase. Mg# shows a flat profile with a strong decrease at the outermost garnet margin. Spessartine component is characterized by generally low values and flat zoning profile and minor increase at the outermost rim of garnet grains. The flat profile of Mg# and spessartine likely indicates diffusive relaxation of a possible earlier growth zonation during high‐temperature metamorphism, probably close to peak conditions. According to phase equilibrium diagrams, the grossular‐rich annulus and the homogeneous Mg# plateau of garnet and the Na‐rich cores of matrix plagioclase represent the composition at peak conditions of ∼0.9–1.1 GPa and ∼820°C (point ‘1’ in Figure 6 and Figure 11a). These *P–T* estimates are in good agreement with the results of ZiR and 2Fsp thermometry and GASP barometry (Figure 11a). Considering the results of all applied techniques, we propose peak conditions of ∼0.9–1.1 GPa and ∼780–820°C for Ysper paragneiss samples.

The metamorphic peak was followed by a stage of near‐isothermal decompression to ∼0.4–0.7 GPa at still elevated temperatures of ∼800–820°C (point ‘2’ in Figure 6). Biotite and plagioclase rims re‐equilibrated at these conditions and the rocks entered the stability field of cordierite. Maximum values obtained from TiB thermometry of ∼780°C coincide with the estimated temperature from phase equilibrium modelling. In contrast, the lower temperatures (i.e., lower Ti values) are likely due to retrograde alteration indicated by ilmenite needles observed as exsolution in some matrix biotites. Cordierite grew preferentially in the melanosome where garnet–sillimanite–biotite are dominant and also formed a corona around garnet porphyroblasts, which further led to the development of symplectite textures. The decrease of Mg# at the outermost rim of garnet grains is interpreted as retrograde diffusive zoning associated with ongoing Fe–Mg exchange with a competing phase such as biotite or cordierite. The slight increase in Mn is more indicative of a partial resorption of garnet, where Mn is not incorporated into a replacing phase but remains in the garnet structure and accumulates at the edges. For the decrease of grossular component after the annulus, we propose a two‐step explanation. First, only grossular is decreasing, whereas Mg# stays constant (Figure 4a,c) or even slightly increases (Figure 4b). In particular, the latter observation could indicate continued garnet growth during a phase of minor further heating ( ∼10–20°C) but already incipient decompression, which then would lead to a decrease in grossular. According to phase equilibrium diagrams and calculated garnet modes, garnet growth is possible following such a path subsequently to peak conditions (Figure 6e). The second step is then most likely related to diffusive modifications during further decompression and cooling. Alternatively, the zoning profile of grossular at the garnet rim could be explained by diffusion. However, since Fe–Mg diffusion in garnet is typically much faster compared with Ca (e.g., Vielzeuf et al., 2007), the distinct decrease of Mg# rather defines the maximum length‐scale of retrograde diffusion (‘o.r.’ in Figure 4a–c). This makes a purely diffusive explanation of the decreasing grossular component at garnet rims unlikely in favour of the proposed two‐step process.

The trace element profiles of garnet (Figure 5) can also be well related to the described *P–T* path. High Y + HREE contents of garnet cores follow a typical Rayleigh fractionation trend where the more compatible HREE were partitioned into garnet during initial crystallisation. Low contents of LREE and MREE indicate that a competitor phase like a phosphate (e.g., monazite and apatite) was stable at this time (e.g., Rubatto et al., 2006; Warren et al., 2018). Along with Ca, also Ti and LREE and MREE (‐Dy) increase. Titanium typically substitutes for Si or Al (Ackerson et al., 2017) and is often interpreted to indicate an increase of *P* and *T*. Unlike the HREE distribution, the LREE and MREE peaks toward the rim cannot be explained by a Rayleigh fractionation model. The rise of LREE and MREE is probably related to the breakdown of accessory minerals such as phosphates (e.g., monazite and apatite) or titanite along the prograde path. Skora et al. (2006) proposed a model in which such REE patterns are controlled by intergranular diffusion rates, which limits the uptake of LREE and MREE at lower temperatures. Depending on the overall concentration in the matrix, the higher diffusion rates with increasing temperature result in the incorporation of greater amounts of LREE and MREE into garnet.

#### 8.1.2 Pielach paragneiss

Pielach valley paragneisses exhibit large, inclusion‐rich garnets with an almost homogeneous chemical composition in most major elements, but an irregular and patchy distribution of Ca, Y and REE (Figure 4 and Figure 5). Mg# and spessartine show similar behaviour to garnet in Ysper samples with a flat profile along the grain followed by a steep decrease or increase at the outermost rim (‘o.r.’ in Figure 4d,e). This again suggests diffusive relaxation of a possible earlier growth zoning during high‐temperature metamorphism. While the decrease in Mg# can be attributed to retrograde diffusive exchange with another Fe‐Mg phase, the increase in Mn is more likely due to garnet resorption. The patchy Ca‐zoning can sometimes be related to wide cracks. Therefore, it could possibly be interpreted as a retrograde feature related to garnet resorption and element exchange with matrix phases. Besides cracks, patchy zoning is also observed in areas with high density of quartz inclusions. This may reflect original quartz‐rich areas in the former matrix, which was then overgrown by garnet, resulting in Ca‐poor domains with high abundance of unreacted quartz inclusions. In plagioclase‐rich areas, however, the overgrown garnet has incorporated larger amounts of Ca, and most of the plagioclase have degraded. Despite the high temperature during metamorphism, the sluggish diffusion of Ca could not compensate for this inhomogeneous distribution. The heterogeneous distribution of trace elements and REE and abundant tiny mineral inclusions do not allow a detailed interpretation of the obtained profiles (Figure 5). A Rayleigh fractionation trend can be observed for Y and some REE, indicating increased incorporation of these elements during initial garnet growth.

Kyanite and rutile inclusions occur almost throughout the entire garnet grain and indicate elevated pressure conditions during garnet growth. Sillimanite joins the inclusion assemblage of kyanite and rutile (Figure 3e) only at the outer garnet rims, suggesting that the metamorphic peak lies close to the sillimanite–kyanite transition within the stability field of rutile. Thermodynamic modelling suggests peak conditions of ∼0.85–1.05 GPa and ∼760–800°C represented by garnet rim composition and related biotite and plagioclase inclusions. In general, these conditions are in good agreement with the results obtained by GASP barometry as well as ZiR and 2Fsp thermometry (Table 4 and Figure 11b). Although the pressure determined by the GASP barometer is slightly higher compared with thermodynamic modelling, they still match quite well. Results of ZiR thermometry show a spread from ∼700 to ∼810°C. While the higher values may represent peak conditions, the lowest may indicate the temperature of the first appearance of rutile along the prograde *P–T* path. By compiling all results, we propose peak metamorphic conditions of ∼0.85–1.1 GPa and ∼760–810°C for Pielach paragneiss samples.

The presence of rutile needles with a preferred orientation, as observed in some garnet porphyroblasts (Figure 2f), is often interpreted as exsolution of rutile from garnet during exhumation (e.g., Ague & Eckert, 2012; Ye et al., 2000; Proyer et al., 2013) and is typically associated with earlier UHP and/or UHT conditions. However, experimental studies on Ti solubility in garnet (Ackerson et al., 2017) have shown that already at *P–T* conditions comparable with our proposed peak conditions, significant amounts of Ti can be incorporated into the garnet lattice. Therefore, and since there is no further evidence for significantly higher *P* or *T* conditions, the mere presence of rutile needles is not a compelling argument for UHT or UHP conditions in the rocks of the Loosdorf complex.

Cordierite is less abundant compared with Ysper samples. It appears only as discontinuous coronae around garnet and as isolated matrix grains intergrown with sillimanite. Although the cordierite composition could not be directly used for *P–T* determination, it can be related to the retrograde stage at ∼0.4–0.5 GPa and ∼750–800°C constrained by thermodynamic modelling and TiB temperatures obtained from matrix biotite (Figure 11b).

### 8.2 Formation of retrograde textures

The formation of cordierite coronae and cordierite–spinel symplectites is a common feature of granulite facies aluminous metasediments (e.g., Carson et al., 1997; Clarke & Powell, 1991; Fitzsimons, 1996; Greenfield et al., 1998; Pitra & De Waal, 2001; Waters, 1991; White et al., 2003). Such textures are usually interpreted as result of retrograde garnet + sillimanite consuming reactions due to near‐isothermal decompression. Typically, an aluminosilicate phase is surrounded by a spinel–cordierite symplectite and an outer cordierite rim, which partly includes quartz. In our samples the (im)mobility of chemical components led to the formation of symplectites along former garnet–sillimanite interfaces involving cordierite–spinel ± corundum ± anorthite ± biotite ± ilmenite on the sillimanite side and cordierite–quartz on the garnet side of the reaction texture. Cordierite textures are absent where garnet is in the proximity of silica‐rich minerals such as feldspars or quartz, indicating the requirement of a locally silica‐undersaturated environment. Consistent with our estimates, the temperature was insufficient to stabilize the potential UHT assemblage of spinel + quartz.

The formation of the observed textures is consistent with minor near‐isothermal decompression. Melt‐depletion and a coarse‐grained peak assemblage, combined with relatively fast exhumation, likely caused the limited transport of strongly‐bonded elements, in particular Al_2_O_3_ and SiO_2_. Monomineralic coronae of cordierite on garnet do not require extensive element exchange, but further hamper transport and facilitates the formation of additional compositional domains. Where other reactions partners such as sillimanite were absent, or outside the length‐scale of transport, texture formation terminated with the development of cordierite coronae. Whereas we did not explicitly consider CaO in our chemical potential calculations for simplicity, its addition would merely stabilize anorthite on the product side where garnet is a reactant, with grossular being the only other, complementary, CaO‐bearing phase. As such, the localised presence of anorthite in the symplectites is consistent with minor CaO diffusing out of garnet, toward sillimanite.

Thermodynamic modelling marks the onset of cordierite growth, which eventually led to the formation of the observed reaction textures, at ∼0.5–0.7 GPa. Although the increased Ca content of the plagioclase matrix rims suggests elevated temperatures, the exact trajectory of retrograde decompression cannot be constrained solely by phase equilibrium modelling and thermobarometry. The first step of texture formation, the growth of the cordierite corona, is at least initially, controlled by the bulk composition of the rock (Figures 6 and 7). However, once the cordierite corona reaches a critical thickness, further development of reaction textures is dictated by chemical potential gradients across local compositional domains, making phase equilibrium diagrams inapplicable. The calculated chemical potential diagrams confirm that the growth of the cordierite corona can be explained by decompression at peak temperature, without requiring cooling (Figure 8). Additional decompression to ∼0.4 GPa then led to the second step of texture formation, the growth of cordierite–spinel and cordierite–quartz symplectites, within local compositional domains defined by cordierite coronae between garnet and sillimanite. Although such chemical potential diagrams cannot be used to precisely constrain metamorphic conditions because of simplifications such as assumptions of a reduced chemical system, they can provide valuable information about the *P–T* trend during mineral texture formation. Nevertheless, temperature conditions can be estimated by the position of the residual solidus at ∼0.4–0.5 GPa to ∼810°C (Figure 6), because crystallisation of the last part of the melt is asserted to be the ideal time of texture formation (White & Powell, 2011). Thus, we conclude that the studied rocks underwent near‐isothermal decompression after the metamorphic peak before switching to cooling at ∼0.4 GPa.

### 8.3 Monazite growth systematics and timing of metamorphism

Both monazite dating methods used in this study yielded identical weighted mean ages (Figure 10). Although individual data from chemical Th–U–total Pb measurements have a larger uncertainty of ∼5–7% compared with isotopic data, analysis by EPMA has the advantage of a better spatial resolution and control on the choice of compositional domains to be analysed. Isotopic U–Pb measurements, on the other hand, provide more precise data, shorter acquisition times and the possibility to assess for discordance and common lead. Several dates, particularly in Ysper samples, show a clear common lead trend and have therefore been omitted from age calculations. However, it is not possible to detect common lead using the EPMA method, which may lead to erroneously old dates. Although the effect of common lead and discordance on individual EPMA dates cannot be addressed, it appears to have little or no effect on the overall data set obtained here, as both Th–U–total Pb chemical dating and U–Pb isotopic dating yield virtually identical weighted mean ages for the investigated samples.

Monazite grains in the matrix of both investigated paragneiss types commonly show Y‐depleted and Th‐rich core and Y‐rich, but Th‐depleted rim domains (Figure 9e,f). No systematic age difference could be obtained between core and rim domains. Both give ages of ∼335 Ma. Monazite inclusions in garnet typically lack these Y‐rich fringes but yielded identical ages. We interpret the Y‐poor core domains of monazite to have grown during prograde metamorphism together with garnet while the formation of Y‐rich fringes is most likely related to the decompression phase. Partial resorption of garnet released some Y, which was then incorporated into newly formed monazite rims (Dumond et al., 2015; Kohn et al., 2005; Pyle & Spear, 2003; Sorger et al., 2020). As the Y‐rich monazite rims did not yield significantly younger ages than Y‐poor monazite cores, the decompression stage is assumed to have started immediately after peak conditions. Monazite in mineral reaction textures also shows distinct Y‐rich fringes like matrix grains. The formation of Y‐rich monazite is therefore interpreted as occurring before or at least simultaneously with the formation of the reaction textures, and the age of ∼335 Ma can be considered as the maximum age for texture formation.

### 8.4 Tectonic interpretation

The Moldanubian zone evolved during the Variscan orogeny as a result of the continental collision of Gondwana and Laurussia. The European Variscan belt exhibits the typical characteristics of a large hot orogen, with considerable ductile flow in the Moldanubian zone. Weakening of the middle and lower crust caused by continuous heating may allow horizontal channel flow or vertical flow of channel material forming dome‐like structures (e.g., Jamieson & Beaumont, 2013). A model of indentation‐driven crustal‐scale buckling and vertical extrusion has been proposed for the exhumation of lower crustal material (e.g., felsic–intermediate granulites) in the eastern Bohemian Massif (Duretz et al., 2011). A transition from vertical to horizontal fabrics described for rocks in the Czech part of the Moldanubian zone favours a two‐stage process with vertical extrusion of lower crustal rocks followed by horizontal channel flow (Schulmann et al., 2008). The first stage of vertical movement caused exhumation of lower crustal rocks and simultaneous burial of middle and upper crustal material, which were finally juxtaposed at middle crustal levels (Štípská et al., 2008). In a second step, these rocks with different *P–T* history were transported and juxtaposed by heterogenous channel flow (Schulmann et al., 2008).

A similar model of vertical channel flow exhumation is proposed for the southeastern part of the Moldanubian zone in Austria. From the data presented here, we conclude that the rocks of the Loosdorf complex experienced Carboniferous high‐grade metamorphism in the middle crust, namely, ∼800°C and 1 GPa ( ∼35 km depth) at ∼340 Ma—consistent with the well‐known coeval regional HT metamorphism in the Moldanubian zone (e.g., Dallmeyer et al., 1992; Friedl, 1997; Friedl et al., 2004; Schulmann et al., 2005; Sorger et al., 2020). Mineral coronae are interpreted in terms of near‐isothermal decompression to ∼0.4–0.5 GPa, implying exhumation to the upper crust ( ∼15 km). Together with the slightly younger weighted mean age of monazite ( ∼335 Ma), taken as the age of a LP–HT overprint and timing of corona formation, we estimate uplift from 35 to 15 km within 5 Myr, yielding a minimum exhumation rate of ∼4 mm·year^−1^. Given the present‐day architecture of the studied complex located in the hanging wall directly above a large Moldanubian granulite body (DWG and PWG in Figure 1), we propose a joint tectonometamorphic history of the two units. In this scenario, the hot granulite body was exhumed from the lower crust to the middle crust where the Loosdorf complex was located and attained the pressure peak reported here. Driven by the high buoyancy of the granulite, both units then moved together to shallower depths, so that the Loosdorf complex reached its final position at an upper crustal level. The voluminous body of hot granulite material allowed exhumation without significant attendant cooling.

The granulite itself, along with the Gföhl gneiss experienced near‐isothermal decompression from ∼1.6 to ∼0.6–0.7 GPa (Figure 11c) before significant cooling occurred (e.g., Carswell & O'Brien, 1993; Cooke & O'Brien, 2001; Schantl et al., 2019). However, these studies focus on the early, high‐temperature evolution of the (U)HT granulites that have experienced significantly higher peak *P–T* conditions. As such, it is possible that the final parts of the exhumation path were not recorded by these rocks, unlike in the texturally complex samples investigated here. Other parts of the Gföhl unit and the Drosendorf unit also experienced near‐isothermal decompression to comparable pressure, but at lower temperature (Petrakakis, 1997; Sorger et al., 2020). In fact, the studied textures allow the interpretation that the Loosdorf complex switched from isothermal decompression to isobaric cooling at ∼0.4–0.5 GPa, within the stability field of cordierite and at considerably lower pressure than other parts of the Gföhl unit.

This scenario would corroborate a short‐lived tectonic event with HT–HP granulite formation followed by a relatively hot exhumation coeval with other parts of the Gföhl unit. The suggested minimum exhumation rate of ∼4 mm·year^−1^ is similar to what has been proposed by other studies of Moldanubian granulites in Austria (e.g., O'Brien & Rötzler, 2003; Schantl et al., 2019) and other granulite occurrences throughout the Bohemian Massif (e.g., Müller et al., 2015; Svojtka et al., 2002; Tajčmanová et al., 2006).

## 9 CONCLUSIONS

- Aluminous paragneisses from the Loosdorf complex experienced MP–HT metamorphism at 0.8–1.1 GPa and 770–820°C, represented by chemically zoned garnet porphyroblasts and associated rutile + sillimanite ± kyanite inclusions. The formation of a cordierite corona and cordierite–spinel or cordierite–quartz textures replacing garnet or sillimanite indicates a LP–HT overprint after near‐isothermal decompression to ∼0.4 GPa and followed by cooling.
- U–Pb and Th–U–total Pb dating of monazite revealed a Carboniferous age of ∼335 Ma. Compared with the widespread ∼340 Ma age for the peak medium‐ to high‐pressure metamorphism, these slightly younger ages are probably related to the LP–HT overprint.
- During the main Variscan collisional phase in the Visean, the Loosdorf rocks were initially buried to ∼35 km depth where they experienced MP–HT metamorphism. The high temperatures were sustained by the exhumation of a lower crustal felsic‐intermediate granulite body transporting high amounts of heat into the middle crust. The coeval exhumation of the Loosdorf complex and the hot granulite facilitated the near‐isothermal decompression to high crustal levels of ∼15 km.

## CONFLICT OF INTEREST STATEMENT

The authors declare that they have no known competing financial interests or personal relationships that could have appeared to influence the work reported in this paper.

## Supporting information




Table S1.

Table S2.
